# A novel role for *STOMATAL CARPENTER 1* in stomata patterning

**DOI:** 10.1186/s12870-016-0851-z

**Published:** 2016-08-02

**Authors:** Giulia Castorina, Samantha Fox, Chiara Tonelli, Massimo Galbiati, Lucio Conti

**Affiliations:** 1 Dipartimento di Bioscienze, Università degli studi di Milano, Via Celoria 26, 20133 Milan, Italy; 2 Department of Cell and Developmental Biology, John Innes Centre, Norwich, NR4 7UH UK

**Keywords:** Arabidopsis, *SCAP1* (AT5G65590), Guard Cells development, DOF-type transcription factors, *SPCH* (AT5G53210), *AtMYB60* (AT1G08810)

## Abstract

**Background:**

Guard cells (GCs) are specialised cells within the plant epidermis which form stomatal pores, through which gas exchange can occur. The GCs derive through a specialised lineage of cell divisions which is specified by the transcription factor *SPEECHLESS* (*SPCH*), the expression of which can be detected in undifferentiated epidermal cells prior to asymmetric division. Other transcription factors may act before GC specification and be required for correct GC patterning. Previously, the DOF transcription factor *STOMATAL CARPENTER 1* (*SCAP1*) was shown to be involved in GC function, by activating a set of GC–specific genes required for GC maturation and activity. It is thus far unknown whether SCAP1 can also affect stomatal development.

**Results:**

Here we show that *SCAP1* expression can also be observed in young leaf primordia, before any GC differentiation occurs. The study of transgenic plants carrying a *proSCAP1:GUS-GFP* transcriptional fusion, coupled with qPCR analyses, indicate that *SCAP1* expression peaks in a temporal window which is coincident with expression of stomatal patterning genes. Independent *scap1* loss-of-function mutants have a reduced number of GCs whilst *SCAP1* over expression lines have an increased number of GCs, in addition to altered GC distribution and spacing patterns. The study of early markers for stomatal cell lineage in a background carrying gain–of–function alleles of *SCAP1* revealed that, compared to the wild type, an increased number of protodermal cells are recruited in the GC lineage, which is reflected in an increased number of meristemoids.

**Conclusions:**

Our results suggest an early role for *SCAP1* in GC differentiation. We propose that a function of *SCAP1* is to integrate different aspects of GC biology including specification, spacing, maturation and function.

**Electronic supplementary material:**

The online version of this article (doi:10.1186/s12870-016-0851-z) contains supplementary material, which is available to authorized users.

## Background

Guard cells (GCs) are specialised epidermal cells which form stomatal pores, through which gas exchange can occur. Since transpiration is linked to plant growth and survival, control of GC number, distribution and activity is tightly regulated. Mature GC pairs form in the epidermal cell layer and originate from a single undifferentiated protodermal cell (PDC). Each PDC undergoes a series of cell divisions and successive cell-state transitions. These transitional states are characterized by changes in cell morphology and are associated with alterations in the transcriptomic signature [1–3]. A subset of PDCs, termed meristemoid mother cells – MMCs –, become competent to initiate the stomatal cell lineage. The MMCs divide asymmetrically to produce a small triangular cell, the meristemoid, which serves as precursor of stomata guard cells and a larger cell referred to as the stomatal lineage ground cell (SLGC). The SLGC has the potential to directly differentiate into a lobed pavement cell or alternatively, to divide again asymmetrically to produce satellite meristemoids. All new meristemoids are oriented at least one cell away from an existing meristemoid according to the one-cell-spacing rule [3–7]. After up to three rounds of amplifying divisions, meristemoids mature into guard mother cells (GMC) acquiring the distinct rounded shape. A GMC divides symmetrically to generate two paired guard cells, which form the stomata pore. The genes responsible for GC specification and development have been characterised: the bHLH-type transcription factors (TFs) *SPEECHLESS* (*SPCH*), *MUTE*, and *FAMA* act sequentially to regulate formation of meristemoids, GMCs and GCs, respectively [8–10]. Alongside the afore-mentioned genes, another class of bHLH–type TFs, *SCREAM/ICE1* and *SCREAM2* redundantly affect the activities of *SPCH*, *MUTE* and FAMA through heterodimerization [11]. Previous studies have shown that *SPCH* is required for cells to enter the stomatal cell lineage and to promote the amplifying divisions of the meristemoids [9, 10, 12]. Experiments utilising *SPCH* promoter-reporter transcriptional fusions revealed that *SPCH* is expressed in the developing leaf epidermis and persists in GMC and GCs throughout the lineage. However, the SPCH protein has only been detected in undifferentiated PDCs, MMCs and in young meristemoids, suggesting that *SPCH* is regulated at the post-transcriptional level [9]. The activity of SPCH protein is negatively regulated by a signalling cascade, which includes secreted peptides EPIDERMAL PATTERNING FACTORS 1 and 2 (EPF1/2), leucine-rich repeat (LRR) receptor-like kinases ERECTA and TOO MANY MOUTHS (*TMM*) [3–5, 7, 13, 14]. The *MITOGEN ACTIVATED KINASE* (*MAPK*) genes act downstream of the LRR receptors and include *YODA*, *MKK4*/*MKK5* and *MPK3*/*MPK6* [15–17]. Stimulation of MAPK results in SPCH phosphorylation and inactivation by proteasomal degradation [1–3, 15, 18].

Several signals converge to regulate the stability of SPCH protein, including the phytohormone Brassinosteroid and CO_2_ [19, 20]. SPCH protein stabilization in protodermal cells is critical to trigger its transcriptional activity and consequent GC lineage entry. Among the direct targets of SPCH is the *EPF2* gene which encodes a peptide that activates a regulatory feed back loop that promotes SPCH protein destabilization [21]. Therefore modulation of SPCH activity translates multiple environmental and endogenous developmental signals into different GC patterns [8–10, 22].

Besides bHLHs, other transcription factors may play an important role in GC specification. The DNA BINDING WITH ONE FINGER (DOF) proteins are an important class of transcriptional regulators in *Arabidopsis thaliana* comprising 37 members [11, 23]. These proteins have been shown to be involved in several aspects of plant development including growth, germination and abiotic stress response [9, 10, 12, 24]. Also, DOF-type factors are implicated in cell cycle control [9, 25]. In stomata development, DOFs have been hypothesized to play a role in GC maturation [3–5, 7, 13, 14, 26, 27]. Recently the DOF transcription factor *STOMATAL CARPENTER 1* (*SCAP1*) has been shown to directly regulate essential processes related to guard cell maturation and function. Mutants of *scap1* display altered levels of transcripts of multiple genes directly involved in stomatal movement and furthermore are defective in some mechanical properties of the GC cell wall [28]. The potential role of *SCAP1* in stomata patterning has not previously been investigated. In this study we provide evidence that *SCAP1* plays a key role in GC patterning, in a manner that is temporally and spatially distinct from its role in GC maturation. We observed *SCAP1* expression throughout the leaf lamina at early developmental stages, when primordia consist of only undifferentiated cells. Mutants of *scap1* had significantly reduced stomatal density and stomatal index compared with wild type. Conversely, over expression of *SCAP1* resulted in increased stomatal density and stomatal index. Furthermore *SCAP1* expression temporarily overlapped with the expression of several other genes that regulate stomatal patterning, consistent with *SCAP1* playing a role in stomata patterning. Induction of SCAP1 activity using a glucocorticoid–based system resulted in repression of several early stomatal patterning genes including *SPCH*, *MUTE* and *EPF2*, and the ectopic production of GCs with altered spacing and morphology. In accordance with these phenotypes, detailed confocal microscopic analysis of marker lines on expanding leaf primordia revealed that high levels of SCAP1 correlated with an increase in the population of meristemoids as well as the number of undifferentiated PDCs. Our work thus provides evidence for a novel role for *SCAP1* in stomatal patterning

## Results

### *SCAP1* expression in leaf precedes GC specification

**Fig. 1:**
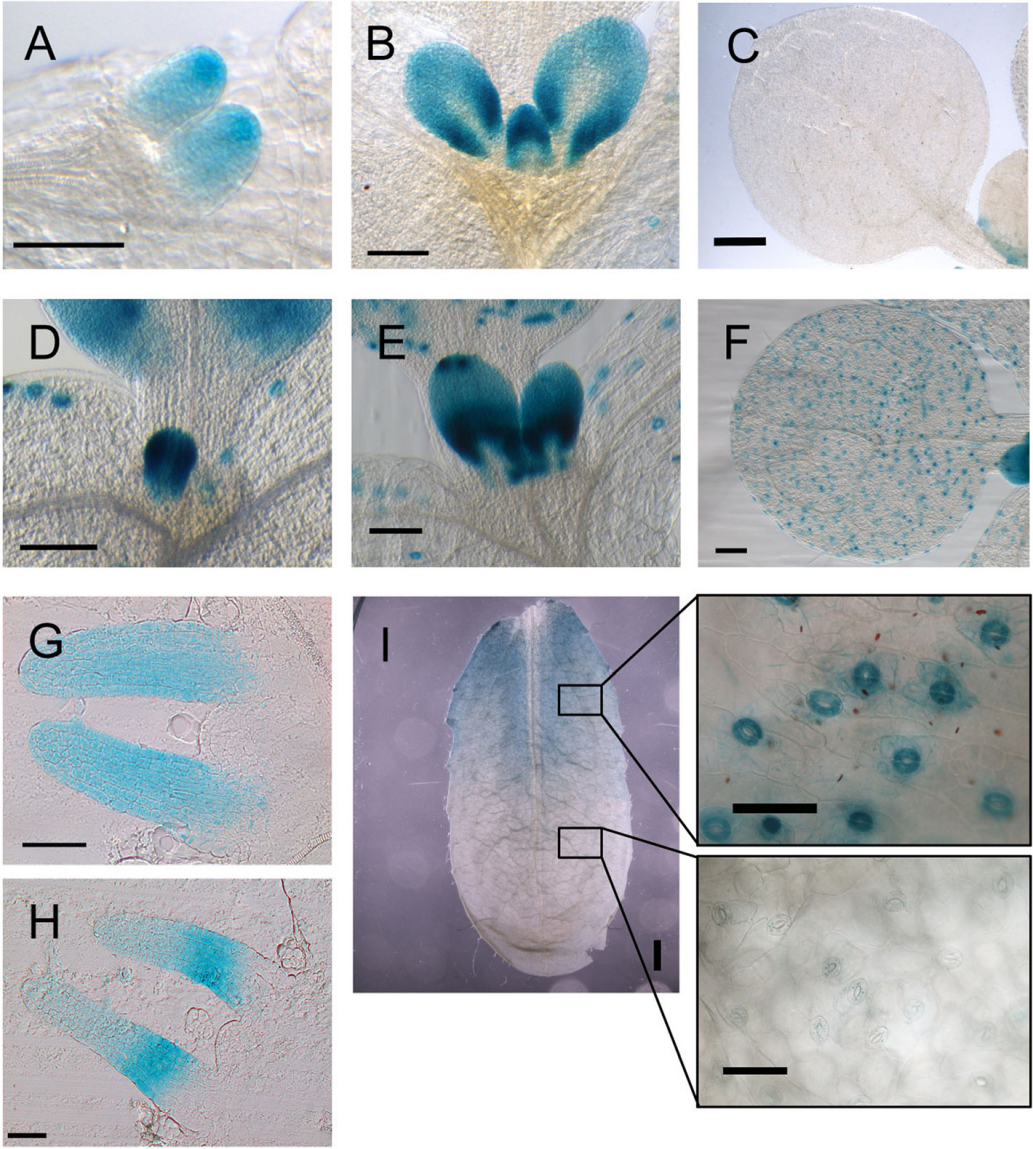
*SCAP1* expression patterns in emerging leaves. (**a**-**c**) GUS staining of the *scap1-2* line and, (**d**-**i**), a representative *proSCAP1:GUS-GFP* transgenic line. Pictures were taken at different stages of leaf development from day 5 (**a**, **d**) to 7 (**b**, **e**). (**g**, **h**) Transversal sections of leaf primordia of a *proSCAP1:GUS-GFP* seedling at day 5, (**g**) and 7 (**h**). (**i**) GCs specific GUS staining at different maturation stages in 3-week-old 6^th^ leaf. Bars = 50 μm (**a**, **b**, **g**, **h**); 500 μm (**c**, **f**); 100 μm (**d**, **e**, **f**); 1 mm (**i**)

To further elucidate the role of *SCAP1* in stomatal development we characterised a *scap1* transposon insertion mutant publicly available in the Cold Spring Harbour collection. This allele (dubbed *scap1-2*) carries a gene trap construct, which permits endogenous patterns of expression of the trapped gene to be visualised via GUS staining. We characterised *scap1-2* plants at different developmental stages and revealed two distinct patterns of gene expression during leaf development (Fig. 1). At early developmental stages (preceding GC formation) GUS staining was present throughout the emerging leaf primordia (Fig. 1a). At later developmental stages of primordia, levels of GUS staining were highest at the flanks of the lamina and much reduced in the midvein region (Fig. 1b). In mature organs (i.e. leaves and cotyledons) the GUS signal was mainly confined to maturing GCs (Fig. 1c). GC–specific *SCAP1* expression was very faint in *scap1-2* mutants compared with transgenic *proSCAP1:GUS-GFP* lines (Fig. 1f) (see below).

The *scap1-2* mutant carries a *GUS* reporter gene in antisense orientation with respect to the *SCAP1* open reading frame (Additional file 1). To verify that the GUS pattern observed in the *scap1-2* allele reflects endogenous *SCAP1* promoter activity we fused a 2977 base pairs genomic region upstream of the *SCAP1* coding sequence to *GUS* and *GFP* and generated independent *Arabidopsis* stable transformants. These transgenic plants (*proSCAP1:GUS-GFP*) displayed GUS activity in young leaf primordia which was similar to that observed in *scap1-2* plants (Fig. 1d, e, f). At later stages of development, the pattern of GUS accumulation in the *proSCAP1:GUS-GFP* lines was broadly similar with that observed in *scap1-2*. Coincident with the expansion of leaf primordia, GUS staining gradually disappeared in the midvein region (Fig. 1e). In young leaf primordia, *SCAP1* promoter activity appeared stronger in the proximal region of the leaf lamina. This observation was confirmed by analysing transverse sections of GUS stained *proSCAP1:GUS-GFP* plants. At early stages of primordium differentiation, the *SCAP1* promoter was uniformly active in the mesophyll and the epidermis of leaf primordia (Fig. 1g). Subsequently we observed a sharp proximodistal gradient of GUS accumulation, with increased signal in the proximal part of the leaf primordium (Fig. 1h). *SCAP1* expression was initially strong in GCs but tended to decrease in a distal to proximal gradient coincident with the maturation of GCs (Fig. 1i). These data reveal a previously undisclosed pattern of *SCAP1* expression in early leaf development, which could suggest an additional role for *SCAP1* alongside its already known function in GC maturation and function.

**Fig. 2:**
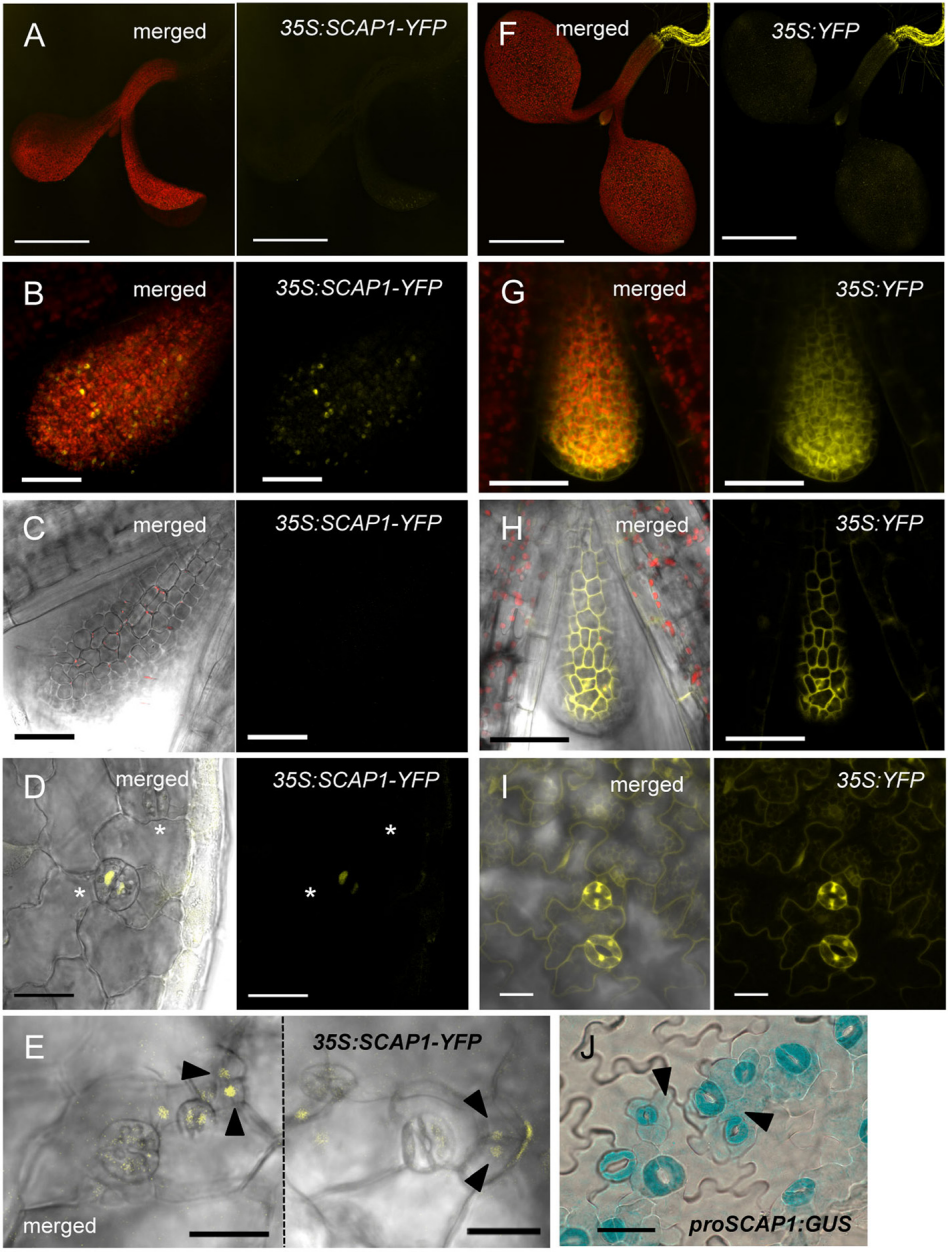
SCAP1 protein differentially accumulates in plant tissues. (**a**-**e**) Confocal images of *pro35S:SCAP1-YFP* (*35S:SCAP1-YFP*) and, (**f**-**i**), *pro35S:YFP* (*35S:YFP*) plants at different stages. (**a**, **f**) Whole seedling. (**b**, **g**) First leaf primordia (5 das). (**c**, **h**) Epidermis of the first leaf primordia (5 das). (**d**, **i**) GCs in a cotyledon (7 das). (**e**) Epidermis of cotyledons (7 das). Asterisks mark epidermal cells, arrows heads mark dividing cells. Images **a**, **b**, **f** and **g** are the sum of all the z stacks obtained across the entire thickness of the sample. Images **c**-**e** and **h**-**i** are the sum of those z stacks corresponding to the epidermis. Bars = 1 mm (**a**, **f**); 50 μm (**b**-**e**, **g**-**i**). SCAP1-YFP/YFP protein signal is shown in yellow, autofluorescence (chlorophyll) in red. (**j**) Cotyledon epidermis of GUS stained *proSCAP1:GUS-GFP* plants (7 das). Bars = 100 μm

To gain insights in SCAP1 protein cellular localization we generated lines of Arabidopsis overexpressing *SCAP1* (*n* = 15). The *SCAP1* coding sequence was fused to the *YELLOW FLUORESCENT PROTEIN* (*YFP*) gene under the control of the constitutive promoter *CaMV35S* (*pro35S:SCAP1-YFP)*. We anticipated that this construct would generate ectopic expression of SCAP1 throughout all plant tissues, however we were only able to observe YFP in a subset of plant tissues. The SCAP1-YFP protein–derived signal was absent in roots (Fig. 2a) whereas control plants overexpressing soluble YFP showed an ectopic signal in all tissues (Fig. 2f-i). We observed SCAP1-YFP accumulation in nuclei of mesophyll cells in young leaf primordia (Fig. 2b), while very little, if any SCAP1-YFP signal was observed in adjacent epidermal cells (Fig. 2c).

At later stages we observed SCAP1-YFP in GCs, which is consistent with the known function of *SCAP1* in GC maturation (Fig. 2d). Detailed analysis of the epidermal layer of *pro35S:SCAP1-YFP* cotyledons revealed low levels of nuclear SCAP1-YFP protein in dividing (or recently divided) epidermal cells adjacent to differentiated GCs (Fig. 2e). In summary, the expression of SCAP1-YFP appeared restricted to the sub-epidermal layer in early leaf primordia and only at later stages of leaf development the expression become visible in mature GCs and adjacent cells. The pattern of SCAP1 protein accumulation at later stages is similar to the domain where the *SCAP1* promoter was transcriptionally active as shown by GUS staining of cotyledons of *proSCAP1:GUS-GFP* plants (Fig. 2j). We conclude that the SCAP1 protein is subject to a strong post-transcriptional regulation and that the site of SCAP1 protein accumulation only partially overlaps with the pattern of *SCAP1* gene expression.

### *SCAP1* regulates GC development

**Fig. 3:**
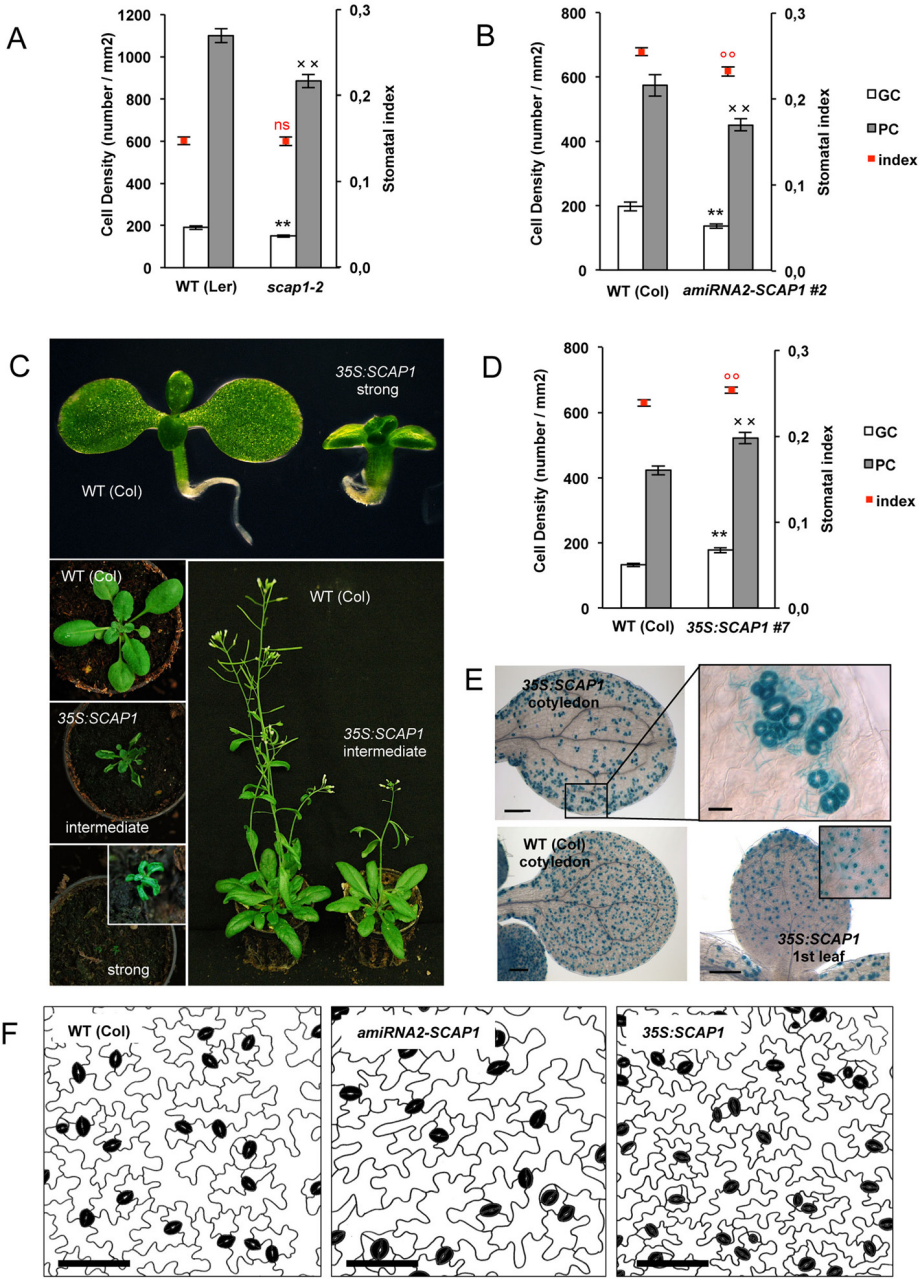
*SCAP1* controls GCs development. (**a**) Number of Guard cells (GC), pavement cells (PC) and stomatal index in wild type (L*er*), and *scap1-2* mutants and, (**b**), wild type (Col) and T3 homozygous *pro35S:amiRNA2-SCAP1* (*amiRNA2-SCAP1*, line #2). (**c**) Morphological alterations observed in *pro35S:SCAP1-YFP* (*35S:SCAP1*) lines at different developmental stages (seedlings, rosette, bolting plants). (**d**) Number of guard cells (GC), pavement cells (PC) and stomatal index in wild type (Col) and a T3 homozygous *pro35S:SCAP1-YFP* (*35S:SCAP1*) intermediate line (line #7). (**e**) GUS staining of double *proAtMYB60:GUS pro35S:SCAP1-YFP (35S:SCAP1)* or single *proAtMYB60:GUS* (WT Col) hemizygous lines. Shown are mature cotyledons (inset, higher magnification of a representative cotyledon area) and the first leaf of 10 days old seedlings. Bar = 200 μm (inset, 25 μm). (**f**) Representative abaxial epidermal phenotype of the 6^th^ expanded leaf of wild type (Col), *pro35S:amiRNA2-SCAP1* (*amiRNA2-SCAP1,* line #2) and *pro35S:SCAP1-YFP* (*35S:SCAP1,* line #7) mutants. Guard cells are false coloured in black. Bar = 50 μm. In **a**, **b**, **d**, (**), (^XX^) and (°°) = P < 0.01 (two tails T Student test) for comparisons between the wild type and the mutant alleles for GC, PC cell density or stomatal index, respectively. ns = not significant. Error bars = Standard Error

The *scap1-2* allele was likely a null since it did not produce any detectable full-length *SCAP1* transcript (Additional file 1). To further investigate the role of *SCAP1* in stomatal development we compared the number of GCs in adult leaves of *scap1-2* mutants with that of wild type (ecotype Landsberg, L*er*). In *scap1-2* stomatal density is reduced (Fig. 3a) but this was not reflected in a reduction of stomatal index since *scap1-2* plants also have a significant reduction in pavement cells compared to wild type (Fig. 3a, Additional file 2). To confirm these observations we generated two independent artificial microRNA (amiRNA1 and 2) constructs specifically targeting *SCAP1* in wild type (ecotype Columbia, Col). We isolated sixteen and fourteen independent T1 lines for *amiRNA1-SCAP1* and *amiRNA2-SCAP1*, respectively and confirmed that T2 lines had reduced levels of *SCAP1* transcript compared to wild type (Additional file 2). Downregulation of *SCAP1* did not produce any obvious phenotypic effects on overall plant morphology, similar to *scap1-2* plants. Closer observations revealed that leaves of segregating T2 *amiRNA-SCAP1* knock-down independent lines produced significantly fewer GCs than wild type (Additional file 2). In homozygous T3 *amiRNA-SCAP1* lines we observed a general reduction in cell density, analogous to the result observed in *scap1-2*, and also a reduction in stomatal index. Taken together these results suggest that *SCAP1* plays a role in GCs specification in addition to its role in cell division (Fig. 3b, f).

To determine whether overexpression of *SCAP1* is sufficient to alter GC development we analysed the phenotypes of the aforementioned *pro35S:SCAP1-YFP* lines. We observed T1 individuals with altered phenotypes ranging in severity from strong to mild (Fig. 3c). Plants classified as strong over-expressors of *SCAP1* (60 %) exhibited numerous developmental defects including reduced germination, slow and stunted growth, upward-curling leaves and sterility. A second phenotypic class (40 %) displayed a less severe phenotype, exhibiting reduced growth compared to wild type at the seedling stage. In transgenic lines with intermediate phenotype, defects appeared to recover at later stages of development so that these lines were eventually comparable in final size and leaf area to wild type. Given the strong phenotypic abnormalities in strong *SCAP1-YFP* overexpressing lines, we carried out our analyses on intermediate lines, which are more comparable to wild type in terms of plant morphology. Lines with intermediate levels of *pro35S:SCAP1-YFP* had an increased number of both GCs and PCs in true leaves compared to wild type and this was accompanied by an overall increase in stomatal index (Fig. 3d, f and Additional file 2). The epidermal phenotype of *pro35S:SCAP1-YFP* plants was characterised in more detail by crossing to a GC–specific reporter line carrying *proAtMYB60:GUS* [29] which allowed us to detect subtler GC patterning defects. The cotyledons of *pro35S:SCAP1-YFP* showed gross alterations in stomata spacing as shown by the presence of massive clusters of GCs which were located at the edges of the cotyledon, especially on the adaxial surface (Fig. 3e). Interestingly, no clusters of GCs were detectable in true leaves of *pro35S:SCAP1-YFP* plants. Furthermore, based on *GUS* detection, over expression of *SCAP1* did not confer guard cell identity to every cell type, nor was it able to induce stomata production in the cotyledon mesophyll cells (Fig. 3e). Thus, *SCAP1* also plays an important role in determining GC spacing, at least in cotyledons.

**Fig. 4:**
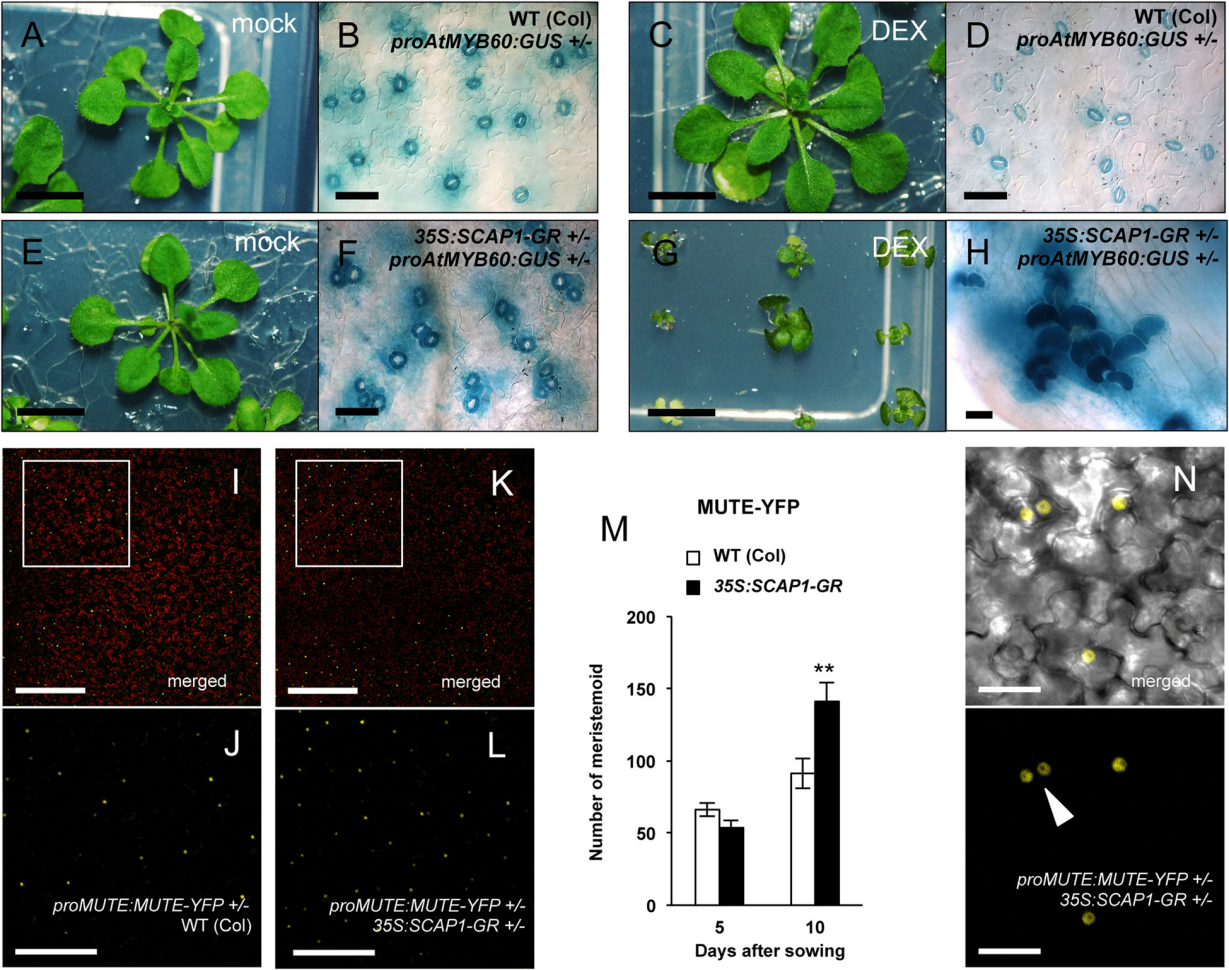
*SCAP1* affect stomata spacing and induce meristemoid production. (**a**-**d**) Morphological alterations observed in 4-weeks old wild type (Col) or (**e**-**h**) *pro35S:SCAP1-GR* (*35S:SCAP1-GR*) plants grown in presence of DEX (**c**-**d** and **g**-**h**) or mock (**a**-**b** and **e**-**f**). (**f**, **h**) GUS staining of double *proAtMYB60:GUS pro35S:SCAP1-GR* or (B, D) single *proAtMYB60:GUS* hemizygous plants. (**i**-**j**) Confocal images of MUTE-YFP fusion proteins in hemizygous *proMUTE:MUTE-YFP* or (**k**-**l** and **n**) double hemizygous *proMUTE:MUTE-YFP pro35S:SCAP1-GR* transgenic plants. Insets (**j** and **l**) are higher magnification of the areas shown in (**i**) and (**k**), respectively. White arrowhead in (**n**) indicates two adjacent meristemoid. (**m**) Number of epidermal cells accumulating MUTE-YFP protein in hemizygous *proMUTE:MUTE-YFP* or double hemizygous *proMUTE:MUTE-YFP pro35S:SCAP1-GR* plants at different developmental stages (5 and 10 das). Shown is the average number of observable nuclei expressing MUTE-YFP in epidermal cells of 10 independent 1^st^ leaf primordia. Note that at stage 5 das, numbers refer to the entire primordium while at 10 das numbers refer to an area of 562 mm^2^. Error bars = Standard Error. ** = P < 0.01 two tails T Student test. Bars = 1 mm (**a**, **c**, **e**, **g**,); 200 μm (**i**, **l**); 100 μm (**j**, **m**); 50 μm (**b**, **d**, **f**); 20 μm (**h**, **o**)

To confirm these observations we generated a second gain-of-function allele of *SCAP1* in which constitutively expressed *SCAP1* is fused to the *GLUCOCORTICOID RECEPTOR* (*pro35S:SCAP1-GR*) [30]. In this inducible system the fusion protein is normally localised in the cytosol but can shuttle to the nucleus upon application of DEXAMETHASONE (DEX) to trigger a rapid SCAP1-dependent transcriptional activation [30]. Prior to induction, plants of *pro35S:SCAP1-GR* were phenotypically indistinguishable from the wild type (Fig. 4e), despite accumulating high levels of *SCAP1-GR* transcript (Additional file 3). *pro35S:SCAP1-GR* seeds did not germinate on media supplemented with DEX, suggesting high levels of *SCAP1* could inhibit germination. Therefore we grew *pro35S:SCAP1-GR* seeds on DEX–free media and transferred seedlings 5 days after sowing on media supplemented with DEX or a mock solution. Twenty days following transfer to DEX *pro35S:SCAP1-GR* plants produced similar morphological alterations previously observed in strong *pro35S:SCAP1-YFP* transgenic plants (Fig. 4g). In contrast, DEX treatment had no significant morphological effects in control plants (Fig. 4c).

To further investigate the epidermal phenotype of *SCAP1–GR* plants we analysed the pattern of GUS distribution in *pro35S:SCAP1-GR proAtMYB60:GUS* double hemizygous plants. Microscopic analysis of untreated *pro35S:SCAP1-GR proAtMYB60:GUS* plants revealed GCs cluster in both cotyledons and leaves although these clusters were generally made of few GCs (Fig. 4f and Additional file 3). Also, *pro35S:SCAP1-GR proAtMYB60:GUS* plants frequently presented unpaired GCs as well as clusters of meristemoid cells adjacent to GCs (Fig. 4f and Additional file 3). DEX treated *pro35S:SCAP1-GR proAtMYB60:GUS* plants, showed an even stronger phenotype in stomata patterning compared with the untreated control as we observed an overproduction of GCs in true leaves, which were grouped in extensive clusters (Fig. 4h). Also in this case, GUS detection revealed that clusters were frequently made of unpaired GCs (Fig. 4h).

To identify whether the altered stomata patterning of *pro35S:SCAP1-GR* could depend on increased number of cells entering the stomatal lineage we generated double hemizygous *proMUTE:MUTE-YFP pro35S:SCAP1-GR* plants which allowed us to visualise meristemoid cells. Even in the absence of DEX, at the later stages of primordium development *pro35S:SCAP1-GR proMUTE:MUTE-YFP* plants displayed an increased number of meristemoids compared to control hemizygous *proMUTE:MUTE-YFP* plants (Fig. 4i to l). A closer inspection of the epidermis revealed that in *pro35S:SCAP1-GR proMUTE:MUTE-YFP* meristemoid cells often did not follow the correct spacing and were close to each other (Fig. 4n). Taken together *SCAP1* appears to regulate different aspects of stomata development, including stomata number, distribution and spacing.

### Effects of *SCAP1* on stomatal patterning gene expression

**Fig. 5:**
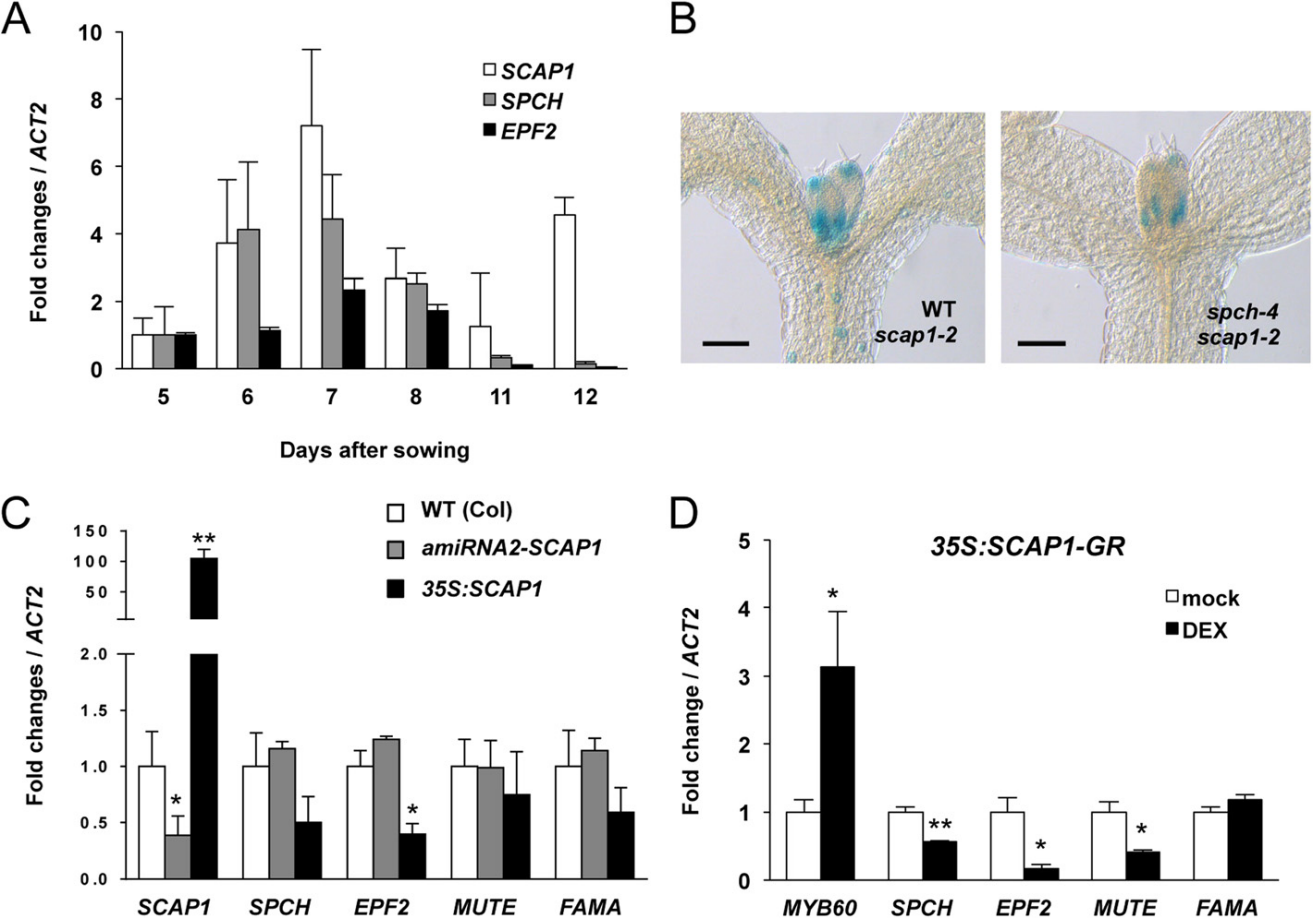
Role of *SCAP1* on stomatal genes transcript accumulations. (**a**) Pattern of *SCAP1*, *SPCH* and *EPF2* transcript accumulations determined by quantitative PCR in manually dissected first two leaf primordia of wild type (Col) seedlings at different days after sowing. Values represent the mean of three biological replicates (30 leaves / replica). (**b**) GUS staining of *scap1-2* in wild type or *spch-4* mutant background in 5 day old seedlings. Bar = 100 μm. (**c**) Pattern of *SCAP1, SPCH*, *EPF2, MUTE* and *FAMA* transcript accumulations determined by quantitative PCR in manually dissected first two leaf primordia of 7 days-old wild type (Col), *pro35S:amiRNA2-SCAP1* (*amiRNA2-SCAP1*) and *pro35S:SCAP1-YFP* (*35S:SCAP1*) plants. Values represent the mean of three biological replicates (30 leaves / replica). (**d**) Pattern of *AtMYB60, SPCH*, *EPF2, MUTE* and *FAMA* transcript accumulations determined by quantitative PCR in 10 days-old *pro35S:SCAP1-GR* (*35S:SCAP1-GR*) plants treated by spraying with DEX (or mock) and the whole seedlings were sampled at eight hours after treatment. Values represent the mean of two biological replicates. In all quantitative PCR *ACTIN* (*ACT2*) was used for normalization. In **c** and **d** ** = P < 0.01 and * = P < 0.05 and two tails T Student test. Error bars = standard deviation

The early activation of *SCAP1* in leaf primordia coupled with its role in stomata development led us to hypothesise a genetic interaction between *SCAP1* and genes that regulate stomatal patterning. Two genes, *SPCH* and *EPF2* that are required for early stomatal patterning are expressed in the protodermal cells of leaf primordia. To determine the timing of *SCAP1* activation with respect to stomata early patterning genes we sampled primordia of leaves one and two from seedlings at different time points, representative of different stages of leaf development. Transcript abundance of *SCAP1*, *SPCH* and *EPF2* peaked at 7 days after sowing and subsequently decreased during the next 3 days (Fig. 5a). At around 12 days after sowing, *SCAP1* expression levels reactivated, presumably in relation to GC formation in the maturing leaf (Fig. 5a). To test if *SCAP1* expression is dependent on *SPCH*, we crossed *scap1-2* (L*er*) with *spch-4* (Col) mutants to obtain homozygous *spch* mutants carrying a transposon tagged version of *SCAP1*. Of 26 *spch* homozygous plants, two displayed GUS staining that was similar in terms of pattern of expression to wild type *SPCH* plants. The reduced frequency of this genotype could be due to genetic linkage since *SPCH* and *SCAP1* are physically close on chromosome 5. *SPCH* is thus not required for the early *SCAP1* activation (Fig. 5b), consistent with previous studies indicating that *SCAP1* was not a high-confidence SPCH target [21].

We next measured transcript accumulation of early stomatal patterning genes in plants with different dosage of *SCAP1*. Transcript levels of *SPCH*, *EPF2*, *MUTE* and *FAMA* were analysed at 7 DAS when levels of *SCAP1*, *SPCH* and *EPF2* expression are at their peak in the wild type (Fig. 5a). In loss-of-function *scap1* mutant plants we detected no significant changes in transcript levels of any of the genes analysed compared with the wild type (Fig. 5c and additional file 4). If *SPCH* and *SCAP1* genetically interact we might predict that increased GC production in *pro35S:SCAP1-YFP* plants would be reflected in an increased level and/or activity of positive regulators of stomatal production, or alternatively down regulation of negative regulators. To determine if this is the case we analysed transcript levels of *SPCH*, *EPF2*, *MUTE* and *FAMA* in the *pro35S:SCAP1-YFP* over expression line. Our analysis confirmed that this transgene conferred around 100-fold increase in *SCAP1* transcript accumulation when compared to wild type (Fig. 5c). Analysis at 7 DAS revealed no significant difference in transcript levels of either *MUTE* or *FAMA* compared to wild type (Fig. 5c). However, we noticed a down regulation of *EPF2* and, marginally, *SPCH* as compared to the wild type (Fig. 5c).

To confirm these observations we analysed the stomatal patterning genes in *pro35S:SCAP1-GR* plants after a short DEX induction. We first tested the ability of SCAP1:GR protein to activate expression of its known target gene *AtMYB60* [28]. Indeed, *scap1* loss of function mutants displayed reduced levels of *AtMYB60* accumulation compared with wild type (Additional file 3). Conversely, compared to wild type plants, *pro35S:SCAP1-GR* plants showed up-regulation of *AtMYB60* after DEX treatment (Fig. 5d and Additional file 3). These data indicate that *SCAP1-GR* protein retains its biochemical function in the context of transcriptional regulation.

Under similar conditions, eight hours after induction we observed a strong downregulation of the negative stomatal regulator *EPF2* (Fig. 5d and Additional file 4). Such *EPF2* downregulation became detectable in DEX treated compared to mock treated plants after four hours and was maintained throughout our experiment (Additional file 4). Besides *EPF2* we also observed a general downregulation of *SPCH* transcript levels and its direct target gene *MUTE*, but not *FAMA* (Fig. 5d). As a control, DEX treatment on wild type plants had no effects in altering stomata patterning genes (Additional file 4). *SCAP1* can therefore act both as a positive and negative transcriptional regulator. However as these experiments were performed on whole seedlings, they may not entirely revel the mode of action of *SCAP1* during the early stages of leaf development.

### *SCAP1* affects SPCH protein accumulation

**Fig. 6:**
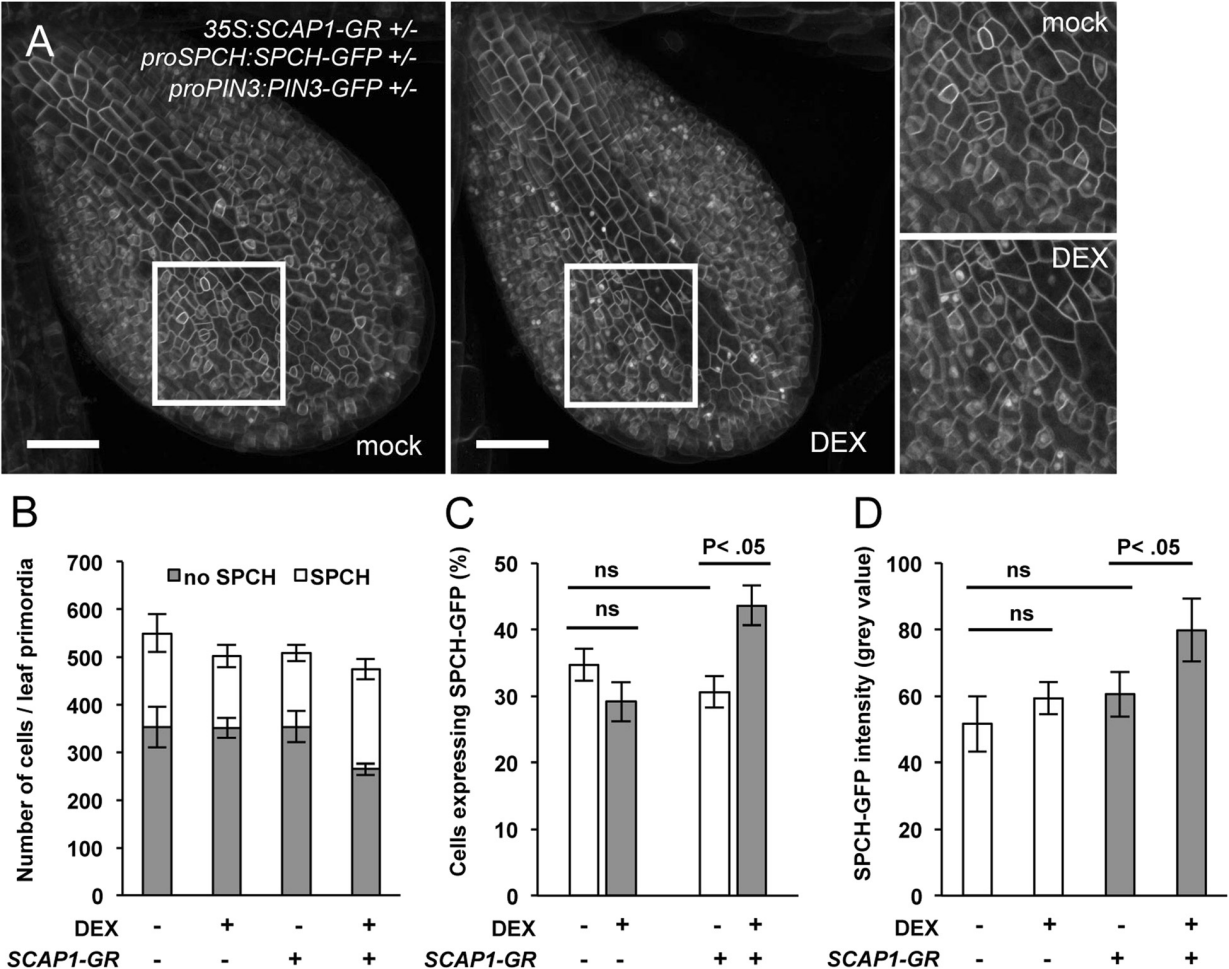
*SCAP1* promotes SPCH protein accumulation. (**a**) Representative picture of the 1^st^ leaf primordia of mock or DEX–treated triple hemizygous *proPIN3:PIN3-GFP proSPCH:SPCH-GFP pro35S:SCAP1-GR* transgenic plants. Insets show a portion of the primordia at higher magnification. PIN3-GFP fusion protein marks the plasma membrane of epidermal cells. SPCH-GFP fusion protein localises in nuclei of the epidermis. Scale bar = 500 μm. (**b** and **c**) Quantification of nuclei accumulating SPCH-GFP in leaf primordia of mock (−) or DEX (+) treated plants. Double hemizygous *proPIN3:PIN3-GFP proSPCH:SPCH-GFP* were used as control (−) and compared to triple hemizygous *proPIN3:PIN3-GFP proSPCH:SPCH-GFP pro35S:SCAP1-GR* (*SCAP1–GR*, +). Bars represent the number of total epidermal cells forming the 1^st^ leaf primordium imaged at 5 das. White bars represent the number of nuclei containing SPCH-GFP. Grey bars are nuclei with no detectable SPCH-GFP. n = 6–8 independent first leaf primordia. This experiment was performed twice with similar results. (**c**) Same data as (**b**) but shown as a percentage of nuclei expressing SPCH-GFP protein over the total number of cell composing leaf primordia (**d**) Quantification of the mean fluorescence intensity of nuclear SPCH-GFP protein in the indicated backgrounds/treatment. Data derived from the analysis of approx. 50 nuclei expressing SPCH-GFP in 6–8 independent first leaf primordia. In (**c**) and (**d**) P values denote a statistical significance in the number of SPCH-GFP nuclei or intensity of SPCH-GFP fluorescence, respectively, calculated with one-way ANOVA. NS = not significant. Error bars = Standard Error

Constitutive expression of *SCAP1* resulted in several developmental abnormalities, which could indirectly alter GC development. To avoid this potential problem, we analysed the effect of SCAP1 after rapid activation by DEX using the *pro35S:SCAP1-GR* line. We studied the pattern SPCH-GFP fusion protein accumulation in the primordia of the first leaf (5 das) through microscope confocal analysis by visualizing nuclear fluorescence of GREEN FLUORESCENT PROTEIN (GFP) in a *proSPCH:SPCH-GFP* line. At 5 das, we did not detect variations in the number of meristemoids, suggesting that SCAP1-GR expression did not yet produce detectable effects at this particular stage (Fig. 4m). We reasoned that by providing a short pulse of *SCAP1* (through DEX applications) we could influence the competence of cells entering the stomata lineage (as estimated by the number of cells expressing SPCH). We generated hemizygous *proSPCH:SPCH-GFP proPIN3:PIN3-GFP pro35S:SCAP1-GR* or hemizygous *proSPCH:SPCH-GFP proPIN3:PIN3-GFP* in a wild type Col background. The PIN3-GFP fusion protein was used as plasma membrane marker and allowed us to identify individual epidermal cells. In control double hemizygous *proSPCH:SPCH-GFP proPIN3:PIN3-GFP* plants no significant differences were found in the number of SPCH-GFP expressing cells following DEX treatment (Fig. 6b and c). Also, DEX treatment did not alter the average intensity of nuclear SPCH-GFP fluorescence, which rules out a general effect of DEX on SPCH-GFP protein accumulation (Fig. 6d). *proSPCH:SPCH-GFP proPIN3:PIN3-GFP pro35S:SCAP1-GR* hemizygous lines showed no apparent defects in SPCH-GFP accumulation at this developmental stage (Fig. 6a to d). At 6 hours following DEX treatment, *proSPCH:SPCH-GFP proPIN3:PIN3-GFP pro35S:SCAP1-GR* plants showed a significant increase in the proportion of nuclei expressing SPCH-GFP protein (Fig. 6a, b and c). Furthermore, this was accompanied with a general increase in the mean nuclear GFP fluorescence intensity (*n* > 50 nuclei / 1^st^ leaf primordia for each genotype/treatment combination) (Fig. 6a, d). It is most likely that the increased nuclear GFP signals reflected increased SPCH-GFP protein since neither DEX treatment or SCAP1-GR alone caused variations in nuclear GFP accumulations (e.g. as a result of detachment of GFP from the membrane marker PIN3 or SPCH). Increased SPCH stabilisation in protodermal cells may thus contribute to stomata pattering alterations in *SCAP1* over expressing plants.

## Discussion

Previously *SCAP1* was shown to control GC morphology and activity, a role coherent with its expression in developing and fully mature stomata [28]. Here we report an in-depth analysis of the spatio-temporal control of *SCAP1* expression throughout leaf development. Our results indicate an early activation of *SCAP1* expression in leaf primordia coinciding with the expression of genes controlling stomatal cell lineage and thus before GC differentiation [6, 7, 10, 31]. This pattern of *SCAP1* gene expression is maintained in *spch* mutants demonstrating that *SCAP1* early expression is independent of GC lineage specification. Besides transcriptional regulation, *SCAP1* is regulated at the post-transcriptional level, as constitutively expressed *SCAP1*-*YFP* fusion did not accumulate in all plant tissues, despite high levels of expression. In leaf primordia where *SCAP1* promoter is active in both epidermis and mesophyll, SCAP1-YFP protein was mainly observed in the mesophyll and in GCs. This observation may either suggest that the role of SCAP1 in GC development is indirect (e.g. to promote signals from the mesophyll cells to the epidermis [32–35] or that the activity of SCAP1 in the epidermis is tightly regulated as a result of rapid protein turn over. Therefore, SCAP1 protein may accumulate in the epidermis in some cell types or at certain stages. Future experiments involving the use of tissue/cell specific promoters to drive *SCAP1* expression may help elucidate the precise cell/tissue-specific pattern of SCAP1 stabilization and provide clues as to the mode of action of *SCAP1* in GC patterning.

A question emerging from our study is whether the role of SCAP1 in stomata patterning is direct or indirect. For example, changes in CO_2_ perceived by one leaf affect the patterning of GCs in subsequent leaves, implying the existence of a signalling network to optimize GC number and patterning according to environmental conditions [36–38]. Since *scap1* mutants are impaired in GC function one could hypothesise that such alterations in stomata activity may affect global GC development. Although we cannot exclude this possibility, we also showed that *SCAP1* overexpressing plants had GC alterations in embryonic tissues such as cotyledons (where we observed GCs cluster). *SCAP1-GR* plants also had increased stomata cells density in true leaves arguing in favour of a direct role of SCAP1 in stomata patterning. Furthermore, a detailed analysis of *SCAP1-GR* plants revealed a role of *SCAP1* in both promoting GCs production and directing the spacing of meristemoid at the very early stages of stomatal cell lineage specification (Fig. 4i to n). These observations are indicative of a role of *SCAP1* in GC patterning which is independent of its general function in GC maturation. The accumulation of *SCAP1* transcript in young leaf primordia is consistent with an early role for *SCAP1* in controlling GC development. *SCAP1* could also play an additional role in the specification of GCs at later stages of leaf development, for example by controlling satellite meristemoid cells, since *SCAP1* expression can be transiently detected in dividing (or recently divided) epidermal cells adjacent to differentiated GCs in older tissue (Fig. 2e).

The overall increased cell density of *SCAP1* overexpressing plants is reminiscent of *SPCH* overexpression or *epf2* mutant plants [6, 7, 9, 15]. Ectopic expression of *SCAP1* could not initiate GCs development in the interior layers of cotyledons or leaves, suggesting that *SCAP1* affects GCs production in conjunction with the known elements of stomatal cell lineage pathway (e.g. *SPCH*, *MUTE* and *FAMA*). Therefore, one attractive hypothesis arising from these observations is that *SCAP1* participates in the same genetic pathway of GC development controlled by *SPCH* and its regulators. Some evidence for this was provided by experiments which showed expression of *SPCH*, *EPF2* and *SCAP1* temporarily overlapping during development. Mutants of *scap1* are not defective in global *SPCH* or *EPF2* gene expression levels, although we have not tested the possibility that the spatial distribution of *SPCH* or *EPF2* genes might be altered in *scap1* mutant plants. Furthermore, the relatively weak epidermal phenotype of *scap1* mutants might be masked by a yet unknown *SCAP1-like* function.

Not only did SCAP1 affect stomata number but also stomata spacing. Clusters of GCs were present in post-embryonic tissues in *pro35S:SCAP1-GR* plants, a phenotype which was even further exacerbated upon DEX application. Surprisingly, this phenotype was not observed in *pro35S:SCAP1-YFP* plants (in which this spacing defects were confined to the cotyledons). The reasons why the *SCAP1-GR* fusion is more active than *SCAP1-YFP* is currently unknown. As SCAP1 accumulation is tightly controlled at the posttranscriptional levels, one possibility is that the GR moiety protects it from degradation.

*SCAP1* overexpression caused *EPF2* downregulation, which could account for spacing defects. The signalling peptides EPF2 acts early in the stomatal lineage controlling asymmetric cell division and thus regulating stomatal density [7]. Previously, comparably similar defects in stomata density and spacing were described in mutants of *epf2, epf1* and *tmm* [3, 5–7, 31], or in transgenic plants overexpressing *SPCH*, *MUTE* or *FAMA* [8, 9, 15]*.* The SPCH protein directly binds and positively regulates the transcription of several stomatal patterning genes including *EPF2*, *MUTE* and *TMM* as well as its own transcription [21]. Our data indicates that some of the direct targets of *SPCH* are negatively regulated by *SCAP1*, suggesting a competition between *SPCH* and *SCAP1* for the regulation of these genes at transcriptional level. In this model, *SCAP1* promotes stomata production and boosts cell divisions by enhancing SPCH protein accumulation possibly as a result of down regulation of *EPF2* transcript. Whether this competition occurs in the same cell and/or is direct should be elucidated by further experiments.

## Conclusions

Our results highlight a previously unappreciated role for *SCAP1* in stomata development. We propose that *SCAP1* is an essential component of a genetic pathway to fine-tune stomatal production in *Arabidopsis*. A key control mechanism of this loop could involve a *SCAP1*-mediated downregulation of *EPF2* counteracting the previously demonstrated SPCH–mediated activation of *EPF2* [21]. DOF-type factors have been proposed to play an important role in GC maturation and function based on an enrichment of a DOF binding motif in GC specific genes [26, 27]. It would be interesting to test whether this observation can also be extended to genes involved in the early events of GC lineage specification. In this sense, *SCAP1* may link GC patterning and function.

## Methods

### Plant material and growth conditions

In this study we used *Arabidopsis thaliana* ecotypes Columbia (Col) and Landsberg *erecta* (L*er*). Seeds were germinated and plants grown in a controlled- environment cabinet at a temperature of 20 °C to 23 °C, 65 % relative humidity, under long day conditions (16 h of light/8 h of dark). Light was cool-white fluorescent tubes (Osram; Sylvania) at a fluency of 120 to 150 μE (photosynthetically active radiation). The *scap1-2* allele is a transposon insertion (line GT-23689, L*er* background) obtained from the Cold Spring Harbour gene trap collection (http://genetrap.cshl.edu). The *spch-4* knock out allele and the *proSPCH:SPCH-GFP proPIN3:PIN3-GFP* and *proMUTE:MUTE-YFP* lines were previously detailed [9, 12, 39]. The *proAtMYB60:GUS* line (Col background) was previously described [29]. The *proSCAP1:GUS-GFP*, *pro35S:amiRNA-SCAP1*, *pro35S:SCAP1-YFP* and *pro35S:SCAP1-GR* lines were generated in this study in wild–type Col background except for the *proSCAP1:GUS-GFP* which was in the L*er* background. Transgenic lines were obtained using the floral dipping method [40]. Transgenic seedlings were selected on Murashige and Skoog (MS) media with kanamycin (50 μg/ml) (*pro35S:SCAP1-GR*) or Basta (25 μM) (*proSCAP1:GUS-GFP*, *pro35S:amiRNA-SCAP1, pro35S:SCAP1-YFP* and *pro35S:SCAP1-GR*). For each construct several T1 independent lines were generated and single insertion transgenic plants were isolated based on the segregation of resistance genes. Independent homozygous T3 lines analysed in this study are : *pro35S:SCAP1-YFP* (#4 and #7), *pro35S:amiRNA-SCAP1* (amiRNA1-2, amiRNA2-2 and amiRNA2-5), *proSCAP1:GUS-GFP* (#8 and #2), *pro35S:SCAP1-GR* (#34 and #26).

Glucocorticoid applications were done by administering a solution of DEX (13 μM DEX, 0.01 % (v/v) Tween 20) either by spraying (for expression analysis) or by soaking seedlings in a MS medium containing DEX (for confocal and GUS experiments). Stratified *pro35S:SCAP1-GR* seeds were germinated on MS plates for 5 to 7 days before spraying with DEX or mock treated or transferred in phytatray (Sigma–Aldrich) on MS media containing DEX for prolonged glucocorticoid treatment.

### Molecular cloning

To generate the *proSCAP1:GUS-GFP* construct a 2977 bp region upstream of the *SCAP1* start codon was amplified from genomic DNA by PCR with oligos attB1-SCAP1 and attB2-SCAP1 which contain the *AttB* adaptors for Gateway–mediated cloning. The PCR product was cloned into *pDONR207* and subsequently transferred to *pBGWFS7* destination vector [41] according to the guidelines detailed in the Gateway protocol (Life Technologies). The *pro35S:amiRNA-SCAP1* constructs were engineered as detailed in http://wmd3.weigelworld.org [42] with primers I, II, III and IV. The PCR products containing *SCAP1*–specific amiRNA were cloned in the pENTR-DTOPO vector (Life Technologies) and transferred to the destination vector pEarleyGate 100 [43] via LR–mediated recombination. To generate the *pro35S:SCAP1-YFP*, the *SCAP1* open reading frame (without stop codon) was amplified by PCR from Arabidopsis DNA, with primers SCAP1-Fw, SCAP1-Re2 and cloned into the pENTR-D TOPO vector (Life Technologies) and recombined with the Gateway destination vector pEarleyGate 101 [43]. The DEX-inducible *SCAP1* construct (*pro35S:SCAP1-GR*) was kindly provided by the RIKEN Plant Functional Genomic Minami Matsui lab. Sequences of the primers are detailed in Additional file 5.

### Genotyping and transcript analysis

Sequences of the primers used for genotyping are provided in Additional file 5. Total RNA was extracted with TRIzol reagent following the manufacturer’s instructions (Life Technologies). The first-strand cDNA was synthesized with 500 ng of total RNA using SuperScript VILO Reverse Transcriptase kit (Life Technologies). Quantitative real-time PCR was performed with Fast SYBR Green Master Mix (Applied Biosystems), and amplification was real-time monitored on a 7900 HT Fast Real-Time PCR system (Applied Biosystems). Changes in gene expression were calculated relative to *ACT2* using the ΔΔCt method [44]. The qPCR primers to detect *SCAP1*, *AtMYB60*, *SPCH*, *EPF2*, *MUTE*, *FAMA* and *ACTIN* transcripts are detailed in Additional file 5.

### β-glucuronidase (GUS) histochemical assay and Histological procedures

GUS staining was performed as previously described [26]. Depending on the experiment, incubation time was for 4 to 12 h at 37 °C. For detection of GUS staining in thin resin sections, after staining, samples were dehydrated in 70 % (v/v) ethanol, post-fixed over night at 4 °C in FAE (50 % [v/v] ethanol, 5 % [v/v] formaldehyde, 10 % [v/v] acetic acid), and further dehydrated in a series of 85 %, 95 % and 100 % (v/v) EtOH and embedded in Technovit 7100 resin according to the manufacturer’s instructions (Heraeus Kulzer). Samples were sectioned with a microtome fitted with a stainless steel blade to a 7 μM thickness.

### Microscopy and quantitative analysis of fluorescence emission

For analysis of the stomatal pattern, the 6^th^ expanded leaves of one-month-old plants (displaying an inflorescence of about 3–4 cm) were incubated in 70 % ethanol. The epidermis of the abaxial side was peeled and examined under a transmission light microscope (DM2500, Leica). For determining the mean stomatal index and density, one square area (0.2 mm^2^) of a leaf region was microphotographed and scored for cell parameters. Care was used to select a similar leaf region from the 6^th^ leaf from at least 12 independent plants for each genotype in independent experiments. For confocal laser scanning microscopy, the abaxial side of first leaf primordia of 5-day-old seedlings expressing GFP- or YFP-tagged proteins were analysed under a Leica TCS SP5 confocal microscope. Fluorochromes were excited using an Argon laser (488 nm and 514 nm excitation for GFP and YFP, respectively) and emission collected at a 500 – 570 nm and 525 – 600 nm for the GFP and YFP, respectively. When comparing independent samples, the acquisition parameters (including z-step size) were maintained constant to enable measurement of GFP intensity in different primordia and different treatments. Fluorescence intensity of nuclear SPCH-GFP protein was analysed with ImageJ software (http://imagej.nih.gov/ij/). GFP fluorescence intensity was measured from individual nuclei (at least 50 for each primordium). A region of interest (ROI) tool was superimposed to selected nuclei so to include the largest possible nuclear area in single optical plane (z stack). An identical ROI size was used to process all the images so to minimise detection of background fluorescence.

## Abbreviations

DEX, Dexamethasone; DOF, DNA Binding With One Finger; GC, Guard cell; GMC, guard mother cell; GR, Glucocorticoid Receptor; MMC, meristemoid mother cell; PC, Pavement cell; PDC, protodermal cell; SLGC, stomatal lineage ground cell; TF, Transcription Factor.

## Additional files

Additional file 1: Characterisation of the *scap1* mutant allele. (A) Schematic representation of the *SCAP1* loci. Grey boxes represent amiRNAs target regions and triangles represent transposon genomic insertion points for *scap1-2*. (B) Reverse Transcriptase-PCR analysis of *SCAP1* in wild type (L*er*) and *scap1-2* plants. Total RNA was isolated from 2-week-old seedlings and PCR was conducted for 35 cycles. Actin was used as a positive control and amplified for 25 cycles. (JPG 97 kb) Additional file 2:
*SCAP1* affects GCs development. (A) Representative abaxial epidermal phenotype of a 6^th^ expanded leaf of wild type (L*er*) and *scap1-2* mutants. Guard cells are false coloured in black. Scale bar = 50 μm. (B) Pattern of *SCAP1* transcript accumulation determined by quantitative PCR in mature leaves in independent T1 BASTA resistant *pro35S:amiRNA-SCAP1* (*amiRNA-SCAP1*) transgenic lines, compared with wild type (Col-0). *ACTIN* (*ACT2*) was used for normalization. Values represent the mean of two technical replicates. Error bars = standard deviation. (C) Number of Guard cells (GC), pavement cells (PC) and stomatal index in wild type (Col) or BASTA selected T2 *pro35S:amiRNA-SCAP1* (*amiRNA-SCAP1*) lines. A transgenic line transformed with empty vector (vector) was used as a further control to account for BASTA treatment. Lines tested in this experiments are labelled in (B) with a filled arrowhead. Line #2, white arrowhead in (B), was not included in this particular experiment. (D) Number of Guard cells (GC), pavement cells (PC) and stomatal index in wild type (Col) or BASTA selected T2 *pro35S:SCAP1-YFP* (*35S:SCAP1*) lines. In C and D (**), (^XX^) and (°°) = P < 0.01 (two tails T Student test) for comparisons between the wild type and the mutant alleles for GC, PC cell density or stomatal index, respectively. ns = not significant. Values of PC in (C) are all not significantly different compared with the wild type or vector. Error bars = Standard Error. (JPG 382 kb) Additional file 3:
*SCAP1* regulates *AtMYB60* expression. (A) *AtMYB60* accumulation determined by quantitative PCR in manually dissected first two-leaf primordia of wild type (WT) *scap1-2* and *pro35S:amiRNA-SCAP1* (*amiRNA2-SCAP1*) seedlings at different time points. Values represent the mean of three biological replicates (30 leaves / replica). (B) *SCAP1* transcript accumulations determined by RT-PCR in *pro35S:SCAP1-GR* T1 lines. Total RNA was isolated from 2-week-old seedlings and PCR was conducted for 30 cycles. Actin was used as a positive control and amplified for 25 cycles. (C) Transcript accumulation of *AtMYB60* determined by quantitative PCR in DEX (or mock) treated wild type (Col) and *pro35S:SCAP1-GR* (*35S:SCAP1-GR*) transgenic plants at different time points. Values represent the mean of two biological replicates. In all quantitative PCR *ACTIN* (*ACT2*) was used for normalization. In A and C, ** = P < 0.01 and * = P < 0.05 and two tails T Student test. Error bars = standard deviation. (D) Morphological alterations of stomata in cotyledons of GUS stained 4-weeks old single *proAtMYB60:GUS* WT (Col) or double *proAtMYB60:GUS pro35S:SCAP1-GR* (*35S:SCAP1-GR*) hemizygous plants. Bar = 20 μm. (E) Close up of GCs surrounded by clusters of meristemoids with altered spacing in cotyledons of DEX treated double *proAtMYB60:GUS pro35S:SCAP1-GR* (*35S:SCAP1-GR*) hemizygous plants (this plant was not subject to GUS staining) Bar = 10 μm. (JPG 370 kb) Additional file 4: Role of *SCAP1* on stomatal genes transcript accumulations. (A) Transcript accumulation of stomatal markers *SPCH*, *EPF2, MUTE* and *FAMA* genes determined by quantitative PCR in manually dissected first two leaf primordia of 7 days-old wild type (L*er*) and *scap1-2* plants. Values represent the mean of three biological replicates (30 leaves/replica). (B-E) Transcript accumulation of stomatal markers *EPF2*, *SPCH*, *MUTE* and *FAMA* genes determined by quantitative PCR in 10 days-old DEX (or mock) treated wild type (Col) and (F-I) *pro35S:SCAP1-GR* (*35S:SCAP1-GR*) transgenic plants at different time point after treatment. Values represent the mean of two biological replicates. In all quantitative PCR *ACTIN* (*ACT2*) was used for normalization. Error bars = standard deviation. ** = P < 0.01 and * = P < 0.05 and two tails T Student test. Values in (A) are all not significantly different compared with the wild type. (JPG 348 kb) Additional file 5: Primers used in this study. (PDF 1736 kb)

## References

[1] Dong J, MacAlister CA, Bergmann DC (2009). BASL controls asymmetric cell division in Arabidopsis. Cell.

[2] Pillitteri LJ, Dong J (2013). Stomatal development in Arabidopsis. Arabidopsis Book.

[3] Geisler M, Nadeau J, Sack FD (2000). Oriented asymmetric divisions that generate the stomatal spacing pattern in arabidopsis are disrupted by the too many mouths mutation. Plant Cell.

[4] Shpak ED, McAbee JM, Pillitteri LJ, Torii KU (2005). Stomatal patterning and differentiation by synergistic interactions of receptor kinases. Science.

[5] Hara K, Kajita R, Torii KU, Bergmann DC, Kakimoto T (2007). The secretory peptide gene EPF1 enforces the stomatal one-cell-spacing rule. Genes Dev.

[6] Hara K, Yokoo T, Kajita R, Onishi T, Yahata S, Peterson KM, Torii KU, Kakimoto T (2009). Epidermal cell density is autoregulated via a secretory peptide, EPIDERMAL PATTERNING FACTOR 2 in Arabidopsis leaves. Plant Cell Physiol.

[7] Hunt L, Gray JE (2009). The signaling peptide EPF2 controls asymmetric cell divisions during stomatal development. Curr Biol.

[8] Ohashi-Ito K, Bergmann DC (2006). Arabidopsis FAMA controls the final proliferation/differentiation switch during stomatal development. Plant Cell.

[9] MacAlister CA, Ohashi-Ito K, Bergmann DC (2007). Transcription factor control of asymmetric cell divisions that establish the stomatal lineage. Nature.

[10] Pillitteri LJ, Sloan DB, Bogenschutz NL, Torii KU (2007). Termination of asymmetric cell division and differentiation of stomata. Nature.

[11] Kanaoka MM, Pillitteri LJ, Fujii H, Yoshida Y, Bogenschutz NL, Takabayashi J, Zhu J-K, Torii KU (2008). SCREAM/ICE1 and SCREAM2 specify three cell-state transitional steps leading to arabidopsis stomatal differentiation. Plant Cell.

[12] Robinson S, Barbier de Reuille P, Chan J, Bergmann D, Prusinkiewicz P, Coen E (2011). Generation of spatial patterns through cell polarity switching. Science.

[13] Hunt L, Bailey KJ, Gray JE (2010). The signalling peptide EPFL9 is a positive regulator of stomatal development. New Phytol.

[14] Lee JS, Kuroha T, Hnilova M, Khatayevich D, Kanaoka MM, McAbee JM, Sarikaya M, Tamerler C, Torii KU (2012). Direct interaction of ligand-receptor pairs specifying stomatal patterning. Genes Dev.

[15] Lampard GR, MacAlister CA, Bergmann DC (2008). Arabidopsis stomatal initiation is controlled by MAPK-mediated regulation of the bHLH SPEECHLESS. Science.

[16] Lampard GR, Lukowitz W, Ellis BE, Bergmann DC (2009). Novel and expanded roles for MAPK signaling in Arabidopsis stomatal cell fate revealed by cell type-specific manipulations. Plant Cell.

[17] Bergmann DC, Lukowitz W, Somerville CR (2004). Stomatal development and pattern controlled by a MAPKK kinase. Science.

[18] Jewaria PK, Hara T, Tanaka H, KONDO T, Betsuyaku S, Sawa S, Sakagami Y, Aimoto S, KAKIMOTO T (2013). Differential effects of the peptides Stomagen, EPF1 and EPF2 on activation of MAP kinase MPK6 and the SPCH protein level. Plant Cell Physiol.

[19] Gudesblat GE, Schneider-Pizoń J, Betti C, Mayerhofer J, Vanhoutte I, van Dongen W, Boeren S, Zhiponova M, de Vries S, Jonak C, Russinova E (2012). SPEECHLESS integrates brassinosteroid and stomata signalling pathways. Nature Cell Biol.

[20] Engineer CB, Ghassemian M, Anderson JC, Peck SC, Hu H, Schroeder JI (2014). Carbonic anhydrases, EPF2 and a novel protease mediate CO2 control of stomatal development. Nature.

[21] Lau OS, Davies KA, Chang J, Adrian J, Rowe MH, Ballenger CE, Bergmann DC (2014). Direct roles of SPEECHLESS in the specification of stomatal self-renewing cells. Science.

[22] Wang H, Ngwenyama N, Liu Y, Walker JC, Zhang S (2007). Stomatal development and patterning are regulated by environmentally responsive mitogen-activated protein kinases in Arabidopsis. Plant Cell.

[23] Riechmann JL, Heard J, Martin G, Reuber L, Keddie J, Adam L, Pineda O, Ratcliffe OJ, Samaha RR, Creelman R (2000). Arabidopsis transcription factors: genome-wide comparative analysis among eukaryotes. Science.

[24] Yanagisawa S (2002). The Dof family of plant transcription factors. Trends Plant Sci.

[25] Skirycz A, Radziejwoski A, Busch W, Hannah MA, Czeszejko J, Kwaśniewski M, Zanor M-I, Lohmann JU, De Veylder L, Witt I, Mueller-Roeber B (2008). The DOF transcription factor OBP1 is involved in cell cycle regulation in Arabidopsis thaliana. Plant J.

[26] Galbiati M, Simoni L, Pavesi G, Cominelli E, Francia P, Vavasseur A, Nelson T, Bevan M, Tonelli C (2008). Gene trap lines identify Arabidopsis genes expressed in stomatal guard cells. Plant J.

[27] Cominelli E, Galbiati M, Albertini A, Fornara F, Conti L, Coupland G, Tonelli C (2011). DOF-binding sites additively contribute to guard cell-specificity of AtMYB60 promoter. BMC Plant Biol.

[28] Negi J, Moriwaki K, Konishi M, Yokoyama R, Nakano T, Kusumi K, Hashimoto-Sugimoto M, Schroeder JI, Nishitani K, Yanagisawa S, Iba K (2013). A Dof Transcription Factor, SCAP1, Is Essential for the Development of Functional Stomata in Arabidopsis. Curr Biol.

[29] Cominelli E, Galbiati M, Vavasseur A, Conti L, Sala T, Vuylsteke M, Leonhardt N, Dellaporta SL, Tonelli C (2005). A guard-cell-specific MYB transcription factor regulates stomatal movements and plant drought tolerance. Curr Biol.

[30] Aoyama T, Chua NH (1997). A glucocorticoid-mediated transcriptional induction system in transgenic plants. Plant J.

[31] Nadeau JA, Sack FD (2002). Control of stomatal distribution on the Arabidopsis leaf surface. Science.

[32] Kondo T, Kajita R, Miyazaki A, Hokoyama M, Nakamura-Miura T, Mizuno S, Masuda Y, Irie K, Tanaka Y, Takada S, KAKIMOTO T, Sakagami Y (2010). Stomatal density is controlled by a mesophyll-derived signaling molecule. Plant Cell Physiol.

[33] Sugano SS, Shimada T, Imai Y, Okawa K, Tamai A, Mori M, Hara-Nishimura I (2010). Stomagen positively regulates stomatal density in Arabidopsis. Nature.

[34] Abrash EB, Bergmann DC (2010). Regional specification of stomatal production by the putative ligand CHALLAH. Development.

[35] Lee JS, Hnilova M, Maes M, Lin Y-CL, Putarjunan A, Han S-K, Avila J, Torii KU (2015). Competitive binding of antagonistic peptides fine-tunes stomatal patterning. Nature.

[36] Lake JA, Quick WP, Beerling DJ, Woodward FI (2001). Plant development: signals from mature to new leaves. Nature.

[37] Lake JA, Woodward FI, Quick WP (2002). Long‐distance CO2 signalling in plants. J Exp Bot.

[38] Coupe SA, Palmer BG, Lake JA, Overy SA, Oxborough K, Woodward FI, Gray JE, Quick WP (2006). Systemic signalling of environmental cues in Arabidopsis leaves. J Exp Bot.

[39] Davies KA, Bergmann DC (2014). Functional specialization of stomatal bHLHs through modification of DNA-binding and phosphoregulation potential. Proc Natl Acad Sci U S A.

[40] Clough SJ, Bent AF (1998). Floral dip: a simplified method for Agrobacterium-mediated transformation of Arabidopsis thaliana. Plant J.

[41] Karimi M, Inzé D, Depicker A (2002). GATEWAY™ vectors for Agrobacterium-mediated plant transformation. Trends Plant Sci.

[42] Ossowski S, Schwab R, Weigel D (2008). Gene silencing in plants using artificial microRNAs and other small RNAs. Plant J.

[43] Earley KW, Haag JR, Pontes O, Opper K, Juehne T, Song K, Pikaard CS (2006). Gateway-compatible vectors for plant functional genomics and proteomics. Plant J.

[44] Livak KJ, Schmittgen TD (2001). Analysis of relative gene expression data using real-time quantitative PCR and the 2(−Delta Delta C(T)) Method. Methods.

