# Erratum to: A novel role for STOMATAL CARPENTER 1 in stomata patterning

**DOI:** 10.1186/s12870-016-0948-4

**Published:** 2017-01-27

**Authors:** Giulia Castorina, Samantha Fox, Chiara Tonelli, Massimo Galbiati, Lucio Conti

**Affiliations:** 1 Dipartimento di Bioscienze, Università degli studi di Milano, Via Celoria 26, 20133 Milan, Italy; 2 Department of Cell and Developmental Biology, John Innes Centre, Norwich, NR4 7UH UK

## Erratum

After publication of this article [1] it was noticed that the picture of a plant that was grown without DEX (and thus intended to be a control), was inadvertently inserted in panel 4C which should represent a DEX-treated plant. The correct version of Fig. 4 can be seen in the next page.

**Fig. 4:**
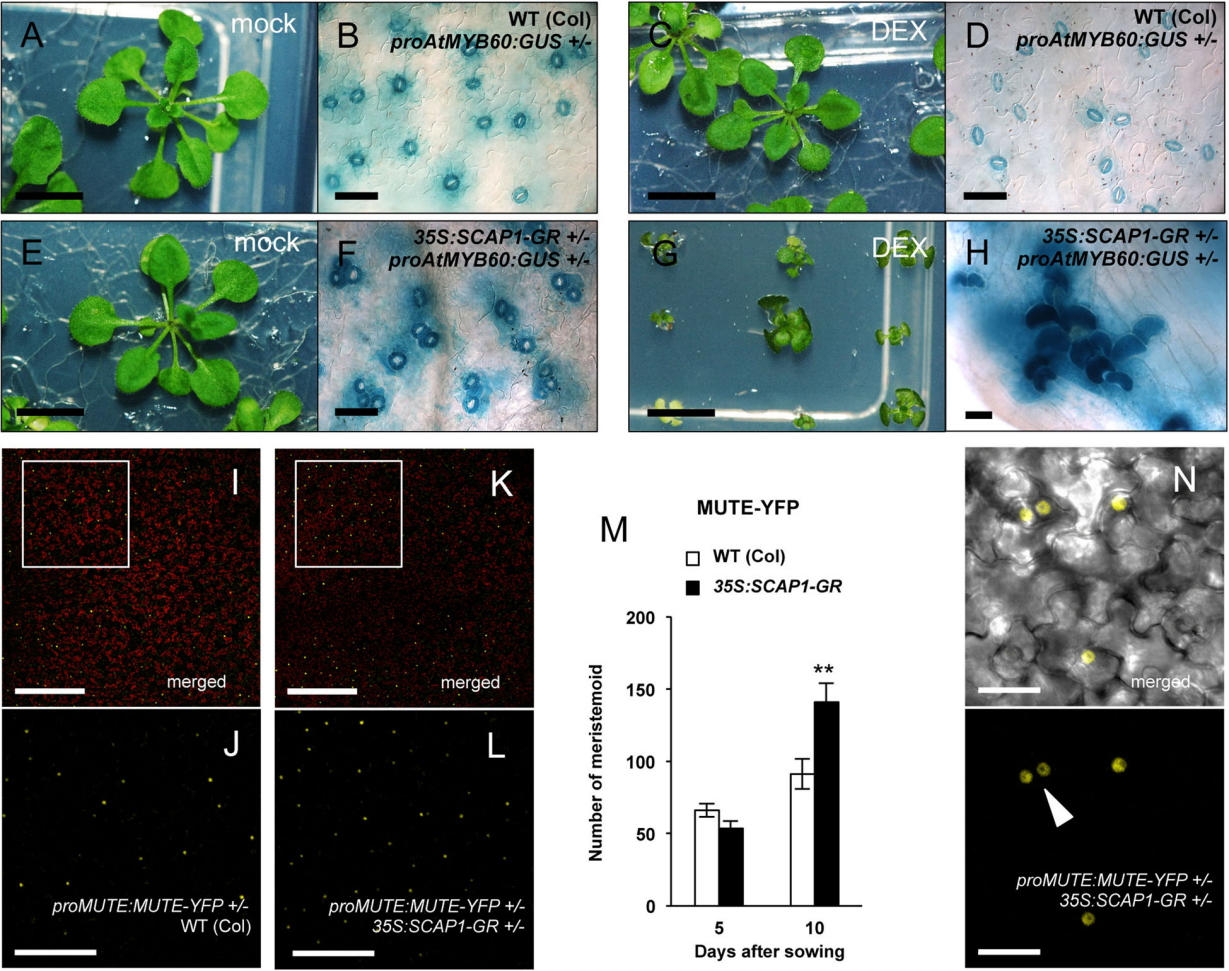
*SCAP1* affect stomata spacing and induce meristemoid production. **a**-**d** Morphological alterations observed in 4-weeks old wild type (Col) or (**e**-**h**) *pro35S*:*SCAP1*-*GR* (*35S*:*SCAP1*-*GR*) plants grown in presence of DEX (**c**-**d** and **g**-**h**) or mock (**a**-**b** and **e**-**f**). **f**, **h** GUS staining of double *proAtMYB60*:*GUS pro35S*:*SCAP1*-*GR* or (B, D) single *proAtMYB60*:*GUS* hemizygous plants. **i**-**j** Confocal images of MUTE-YFP fusion proteins in hemizygous *proMUTE*:*MUTE*-*YFP* or (**k**-**l** and **n**) double hemizygous *proMUTE*:*MUTE*-*YFP pro35S*:*SCAP1*-*GR* transgenic plants. Insets (**j** and **l**) are higher magnification of the areas shown in (**i**) and (**k**), respectively. White arrowhead in (**n**) indicates two adjacent meristemoid. **m** Number of epidermal cells accumulating MUTE-YFP protein in hemizygous *proMUTE*:*MUTE*-*YFP* or double hemizygous *proMUTE*:*MUTE*-*YFP pro35S*:*SCAP1*-*GR* plants at different developmental stages (5 and 10 das). Shown is the average number of observable nuclei expressing MUTE-YFP in epidermal cells of 10 independent 1st leaf primordia. Note that at stage 5 das, numbers refer to the entire primordium while at 10 das numbers refer to an area of 562 mm^2^. Error bars = Standard Error. ** = *P* < 0.01 two tails T Student test. Bars = 1 mm (**a**, **c**, **e**, **g**,); 200 μm (**i**, **l**); 100 μm (**j**, **m**); 50 μm (**b**, **d**, **f**); 20 μm (**h**, **o**)
