# Supplementary figures and images for: A novel role for STOMATAL CARPENTER 1 in stomata patterning
Source: BMC Plant Biol. 2016 Aug 2;16:172. doi: 10.1186/s12870-016-0851-z (PMC4970199; doi:10.1186/s12870-016-0851-z)

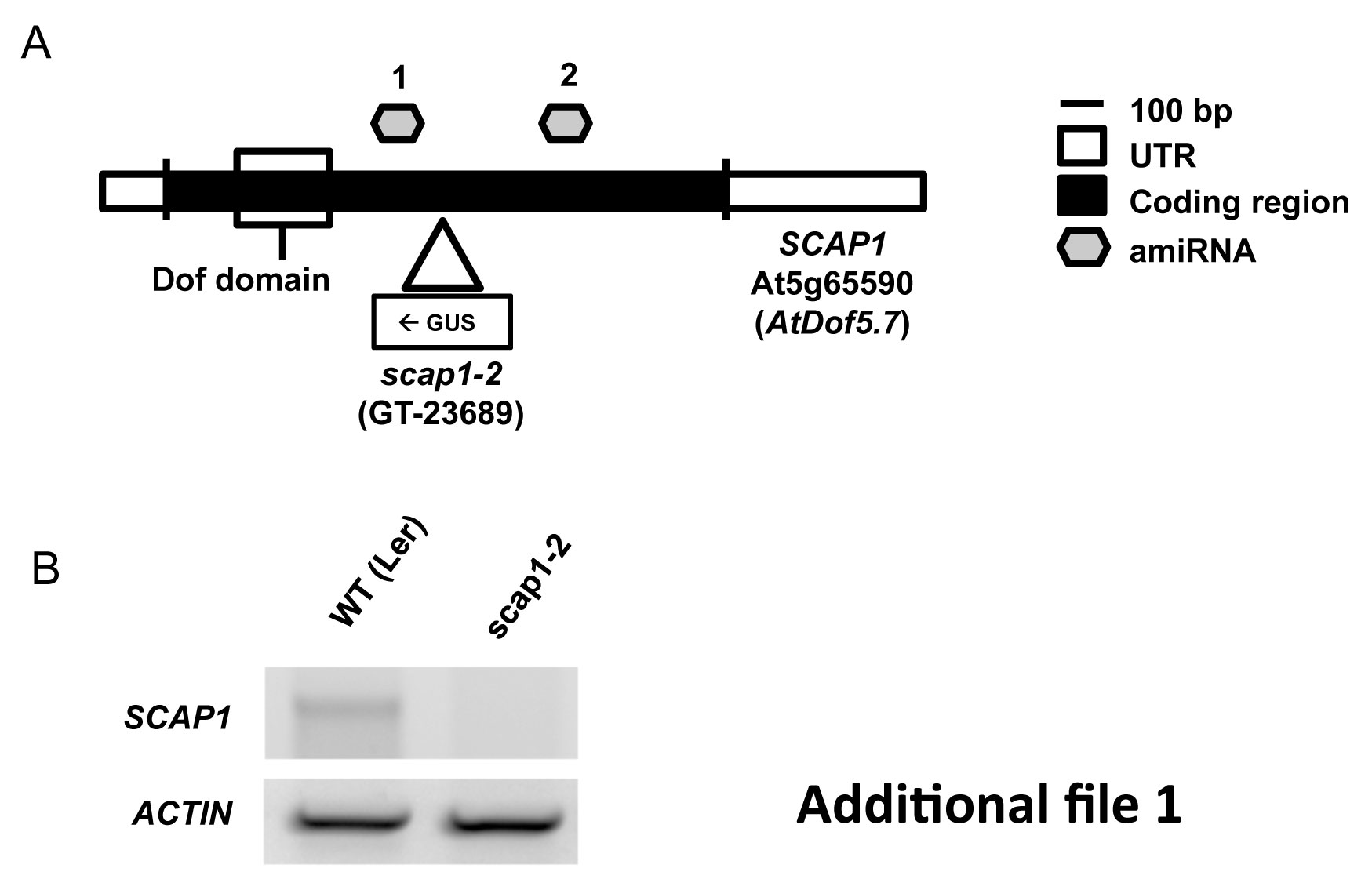

Supplement: Additional file 1: — Characterisation of the scap1 mutant allele. (A) Schematic representation of the SCAP1 loci. Grey boxes represent amiRNAs target regions and triangles represent transposon genomic insertion points for scap1-2. (B) Reverse Transcriptase-PCR analysis of SCAP1 in wild type (Ler) and scap1-2 plants. Total RNA was isolated from 2-week-old seedlings and PCR was conducted for 35 cycles. Actin was used as a positive control and amplified for 25 cycles. (JPG 97 kb) [file 12870_2016_851_MOESM1_ESM.jpg]

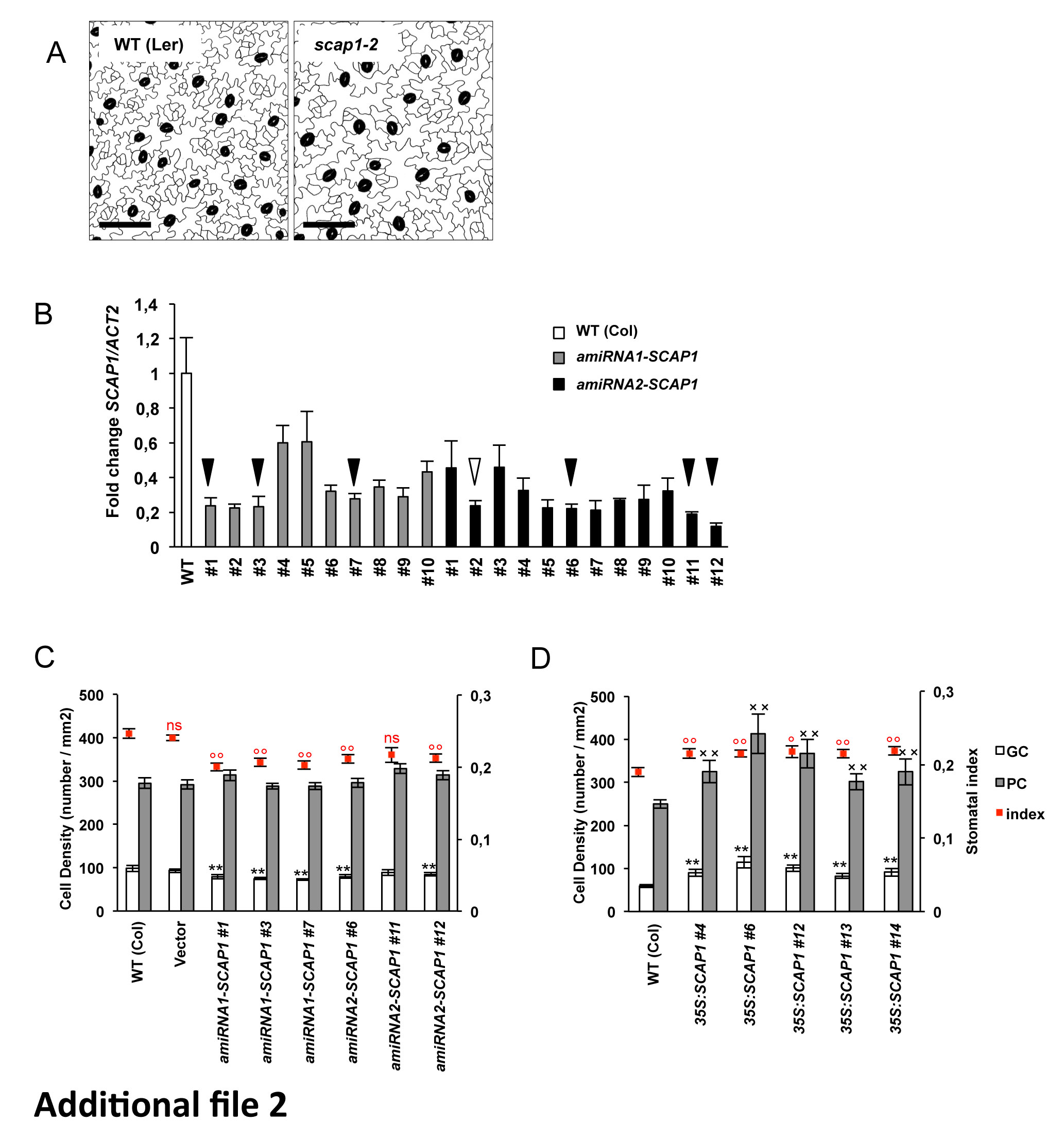

Supplement: Additional file 2: — SCAP1 affects GCs development. (A) Representative abaxial epidermal phenotype of a 6th expanded leaf of wild type (Ler) and scap1-2 mutants. Guard cells are false coloured in black. Scale bar = 50 μm. (B) Pattern of SCAP1 transcript accumulation determined by quantitative PCR in mature leaves in independent T1 BASTA resistant pro35S:amiRNA-SCAP1 (amiRNA-SCAP1) transgenic lines, compared with wild type (Col-0). ACTIN (ACT2) was used for normalization. Values represent the mean of two technical replicates. Error bars = standard deviation. (C) Number of Guard cells (GC), pavement cells (PC) and stomatal index in wild type (Col) or BASTA selected T2 pro35S:amiRNA-SCAP1 (amiRNA-SCAP1) lines. A transgenic line transformed with empty vector (vector) was used as a further control to account for BASTA treatment. Lines tested in this experiments are labelled in (B) with a filled arrowhead. Line #2, white arrowhead in (B), was not included in this particular experiment. (D) Number of Guard cells (GC), pavement cells (PC) and stomatal index in wild type (Col) or BASTA selected T2 pro35S:SCAP1-YFP (35S:SCAP1) lines. In C and D (**), (XX) and (°°) = P < 0.01 (two tails T Student test) for comparisons between the wild type and the mutant alleles for GC, PC cell density or stomatal index, respectively. ns = not significant. Values of PC in (C) are all not significantly different compared with the wild type or vector. Error bars = Standard Error. (JPG 382 kb) [file 12870_2016_851_MOESM2_ESM.jpg]

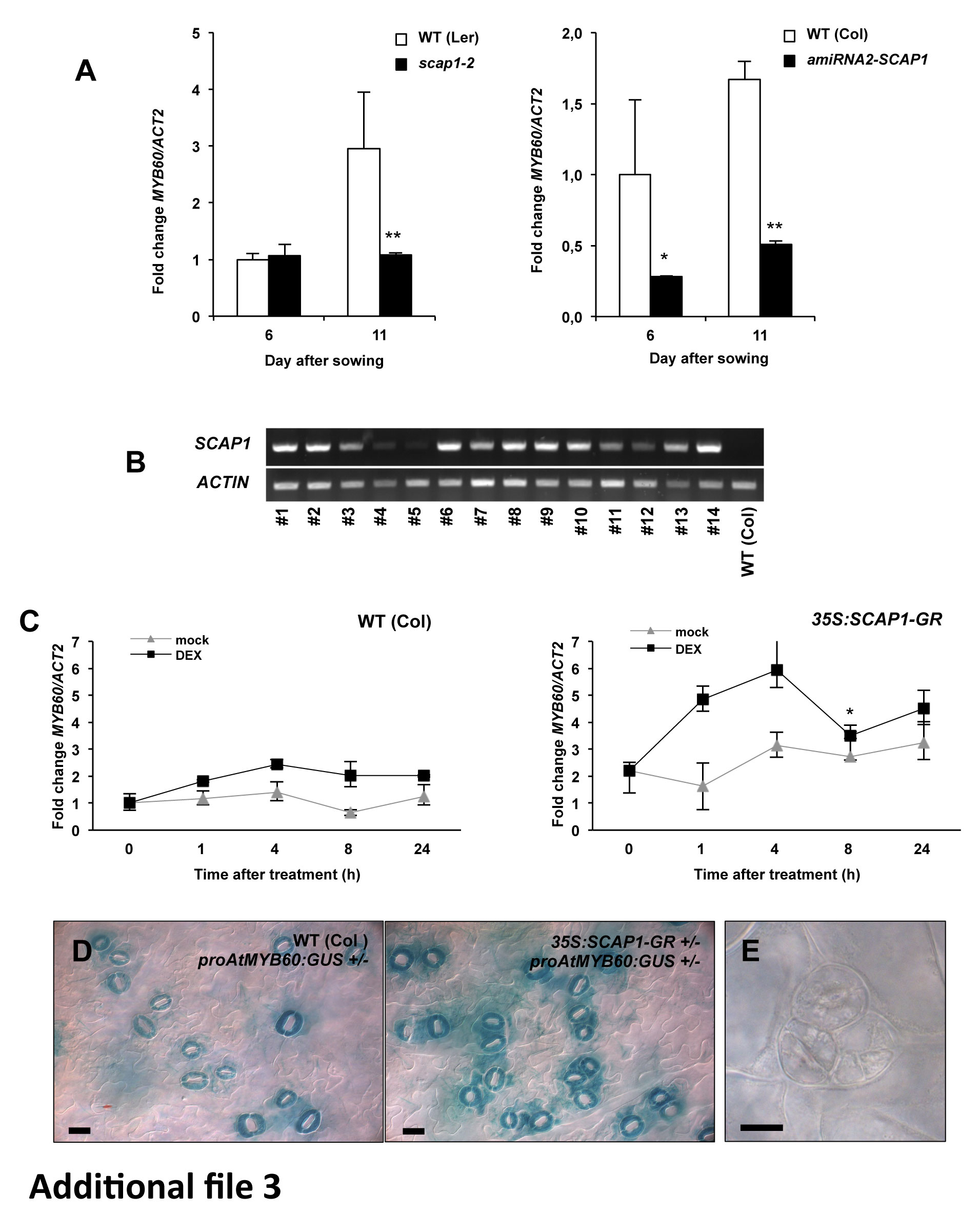

Supplement: Additional file 3: — SCAP1 regulates AtMYB60 expression. (A) AtMYB60 accumulation determined by quantitative PCR in manually dissected first two-leaf primordia of wild type (WT) scap1-2 and pro35S:amiRNA-SCAP1 (amiRNA2-SCAP1) seedlings at different time points. Values represent the mean of three biological replicates (30 leaves / replica). (B) SCAP1 transcript accumulations determined by RT-PCR in pro35S:SCAP1-GR T1 lines. Total RNA was isolated from 2-week-old seedlings and PCR was conducted for 30 cycles. Actin was used as a positive control and amplified for 25 cycles. (C) Transcript accumulation of AtMYB60 determined by quantitative PCR in DEX (or mock) treated wild type (Col) and pro35S:SCAP1-GR (35S:SCAP1-GR) transgenic plants at different time points. Values represent the mean of two biological replicates. In all quantitative PCR ACTIN (ACT2) was used for normalization. In A and C, ** = P < 0.01 and * = P < 0.05 and two tails T Student test. Error bars = standard deviation. (D) Morphological alterations of stomata in cotyledons of GUS stained 4-weeks old single proAtMYB60:GUS WT (Col) or double proAtMYB60:GUS pro35S:SCAP1-GR (35S:SCAP1-GR) hemizygous plants. Bar = 20 μm. (E) Close up of GCs surrounded by clusters of meristemoids with altered spacing in cotyledons of DEX treated double proAtMYB60:GUS pro35S:SCAP1-GR (35S:SCAP1-GR) hemizygous plants (this plant was not subject to GUS staining) Bar = 10 μm. (JPG 370 kb) [file 12870_2016_851_MOESM3_ESM.jpg]

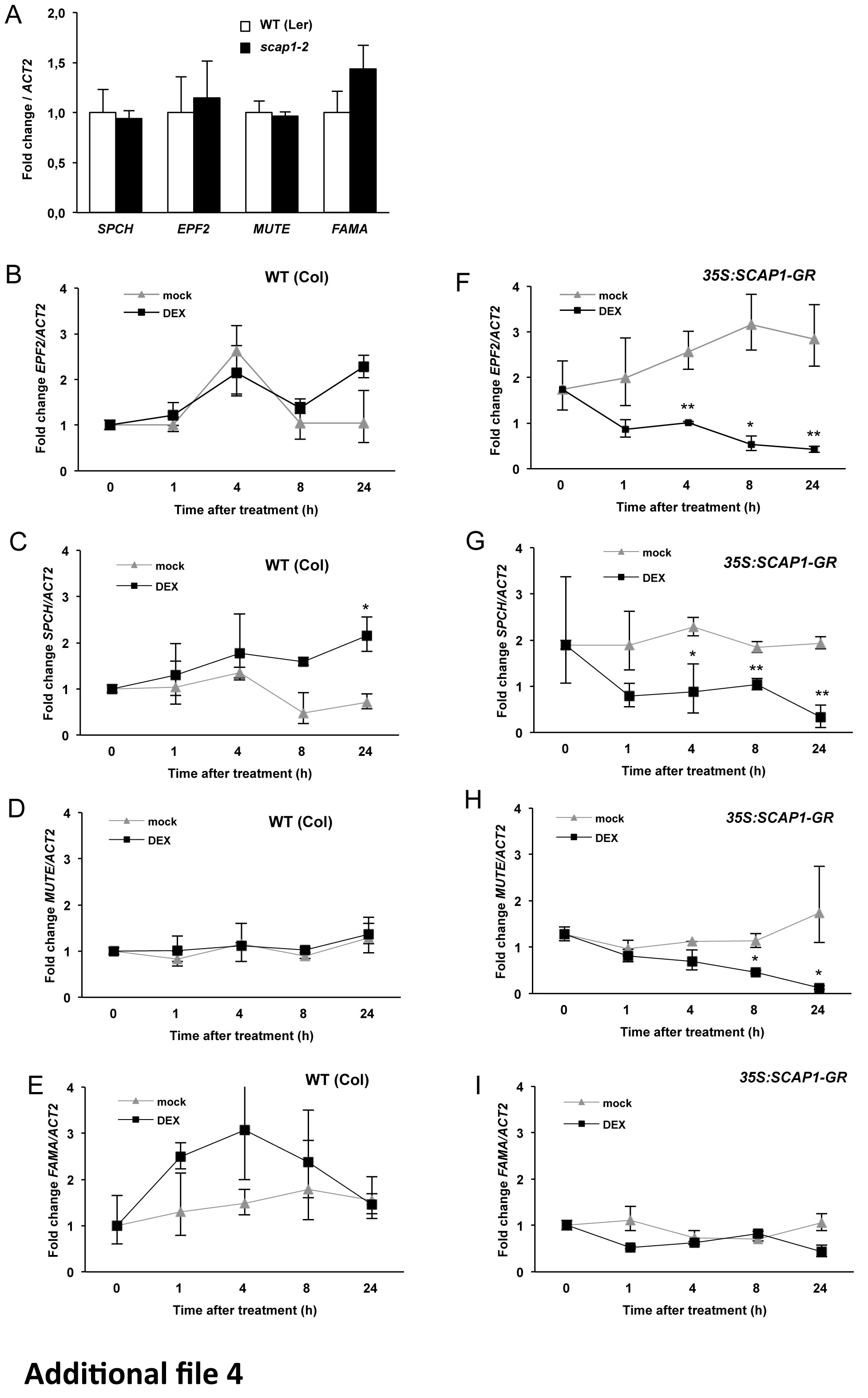

Supplement: Additional file 4: — Role of SCAP1 on stomatal genes transcript accumulations. (A) Transcript accumulation of stomatal markers SPCH, EPF2, MUTE and FAMA genes determined by quantitative PCR in manually dissected first two leaf primordia of 7 days-old wild type (Ler) and scap1-2 plants. Values represent the mean of three biological replicates (30 leaves/replica). (B-E) Transcript accumulation of stomatal markers EPF2, SPCH, MUTE and FAMA genes determined by quantitative PCR in 10 days-old DEX (or mock) treated wild type (Col) and (F-I) pro35S:SCAP1-GR (35S:SCAP1-GR) transgenic plants at different time point after treatment. Values represent the mean of two biological replicates. In all quantitative PCR ACTIN (ACT2) was used for normalization. Error bars = standard deviation. ** = P < 0.01 and * = P < 0.05 and two tails T Student test. Values in (A) are all not significantly different compared with the wild type. (JPG 348 kb) [file 12870_2016_851_MOESM4_ESM.jpg]
