# Supplementary material for: A novel role for STOMATAL CARPENTER 1 in stomata patterning
Source: BMC Plant Biol. 2016 Aug 2;16:172. doi: 10.1186/s12870-016-0851-z (PMC4970199; doi:10.1186/s12870-016-0851-z)
Supplement: Additional file 5: — Primers used in this study. (PDF 1736 kb) [file 12870_2016_851_MOESM5_ESM.pdf]

Additional file 5: Primers used in this study.

Purpose	Primer name	Sequence	Reference
SCAP1 promoter cloning	attB1-SCAP1	AAAAAGCAGGCTTCctctgtttttgtttcatcccaaag	
	attB2-SCAP1	AGAAAGCTGGGTGgtaaagtaggagaggagagagagaac	
amiRNA-SCAP1 cloning	1-I	gaTATTAGGGATAAACGCGACCCtctctcttttgtattcc	
	1-II	gaGGGTCGCGTTTATCCCTAATAtcaaagagaatcaatga	
	1-III	gaGGATCGCGTTTATGCCTAATTtcacaggtcgtgatatg	
	1-IV	gaAATTAGGCATAAACGCGATCCtctacatatatattcct	
	2-I	gaTAATGATTAAGATCGGTCCGActctcttttgtattcca	
	2-II	agTCGGACCGATCTTAATCATTAtcaaagagaatcaatga	
	2-III	agTCAGACCGATCTTTATCATTTtcacaggtcgtgatatg	
	2-IV	gaAAATGATAAAGATCGGTCTGActacatatatattccta	
	A	caccCTGCAAGGGATTAAGTTGGGTAAC	
	B	GCGGATAACAATTTCACACAGGAAACAG	
SCAP1 CDS cloning	SCAP1-Fw	caccATGTCCTCCCATACCAATCTCC	
	SCAP1-Re2	AGGCAATGCATTATAATGATTAAG	
Genotyping RT-PCR	SCAP1-Re3	TCAAGGCAATGCATTATAATGATTAA	
	Ds3-1	ACCCGACCGGATCGTATCGGT	[1]
	SPCH-Fw	AAAACAAATTCGTTTGCTCCC	[2]
	SPCH-Re	TATGAGGGACTCGCATTCATC	
	LBb1.3	ATTTTGCCGATTTCGGAAC	
qRT-PCR	qRT-SCAP1-Fw	GCCGACGAAGAAAGGAGATA	
	qRT-SCAP1-Re	TCCAAGCTTGAATTCCATTG	
	qRT-MYB60-Fw	CATGAAGATGGTGATCATGAGG	[3]
	qRT-MYB60-Re	TTCCATTTGACCCCCAGTAG	
	qRT-SPCH-Fw	CGCTTCGCTCTTACAGCTCA	[4]
	qRT-SPCH-Re	CCGTTTTGAGCAGCACGTTA	
	qRT-MUTE-Fw	CAGACAATCGAGCCATCCAG	
	qRT-MUTE-Re	CCCACGATTCGCCTAGAGAC	
	qRT-FAMA-Fw	CTCCTACGTTCAAAGGGGAGAC	[5]
	qRT-FAMA-Re	CTTCGACGCTTCTGTGATTCAAGAC	
	qRT-EPF2-Fw	TTTGGTCGTTAACTCCATTCG	[6]
	qRT-EPF2-Re	ATCCGGTAAGCTTGATCCTGT	
	qRT-ACT-Fw	GTGTTGGACTCTGGAGA TGGTGTG	
	qRT-ACT-Re	GCCAAAGCAGTGATCTCTTTGCTC	

Additional references

1. Galbiati M, Simoni L, Pavesi G, Cominelli E, Francia P, Vavasseur A, Nelson T, Bevan M, Tonelli C: **Gene trap lines identify Arabidopsis genes expressed in stomatal guard cells.** *Plant J* 2008, **53**:750–762.

2. MacAlister CA, Ohashi-Ito K, Bergmann DC: **Transcription factor control of asymmetric cell divisions that establish the stomatal lineage.** *Nature* 2007, **445**:537–540.

3. Cominelli E, Galbiati M, Albertini A, Fornara F, Conti L, Coupland G, Tonelli C: **DOF-binding sites additively contribute to guard cell-specificity of AtMYB60 promoter.** *BMC Plant Biol* 2011, **11**:162.

4. Tanaka Y, Nose T, Jikumaru Y, Kamiya Y: **ABA inhibits entry into stomatal-lineage development in Arabidopsis leaves.** *Plant J* 2013, **74**:448–457.

5. Niwa T, Kondo T, Nishizawa M, Kajita R, Kakimoto T, Ishiguro S: **EPIDERMAL PATTERNING FACTOR LIKE5 peptide represses stomatal development by inhibiting meristemoid maintenance in Arabidopsis thaliana.** *Biosci Biotechnol Biochem* 2013, **77**:1287–1295.

6. Pillitteri LJ, Peterson KM, Horst RJ, Torii KU: **Molecular profiling of stomatal meristemoids reveals new component of asymmetric cell division and commonalities among stem cell populations in Arabidopsis.** *Plant Cell* 2011, **23**:3260–3275.
